# Structural and Evolutionary Insights within the Polysaccharide Deacetylase Gene Family of *Bacillus anthracis* and *Bacillus cereus*

**DOI:** 10.3390/genes9080386

**Published:** 2018-07-31

**Authors:** Athena Andreou, Petros Giastas, Elias Christoforides, Elias E. Eliopoulos

**Affiliations:** 1 Department of Biotechnology, Laboratory of Genetics, Agricultural University of Athens, Iera Odos 75, 11855 Athens, Greece; aandre@aua.gr (A.A.); el_chr@aua.gr (E.C.); 2 Department of Neurobiology, Hellenic Pasteur Institute, Vasilissis Sofias 127, 11521 Athens, Greece; petrosgiastas@gmail.com

**Keywords:** *Bacillus anthracis*, *Bacillus cereus*, polysaccharide deacetylase, functional divergence, structural evolution

## Abstract

Functional and folding constraints impose interdependence between interacting sites along the protein chain that are envisaged through protein sequence evolution. Studying the influence of structure in phylogenetic models requires detailed and reliable structural models. Polysaccharide deacetylases (PDAs), members of the carbohydrate esterase family 4, perform mainly metal-dependent deacetylation of *O*- or *N*-acetylated polysaccharides such as peptidoglycan, chitin and acetylxylan through a conserved catalytic core termed the NodB homology domain. Genomes of *Bacillus anthracis* and its relative *Bacillus cereus* contain multiple genes of putative or known PDAs. A comparison of the functional domains of the recently determined PDAs from *B. anthracis* and *B. cereus* and multiple amino acid and nucleotide sequence alignments and phylogenetic analysis performed on these closely related species showed that there were distinct differences in binding site formation, despite the high conservation on the protein sequence, the folding level and the active site assembly. This may indicate that, subject to biochemical verification, the binding site-forming sequence fragments are under functionally driven evolutionary pressure to accommodate and recognize distinct polysaccharide residues according to cell location, use, or environment. Finally, we discuss the suggestion of the paralogous nature of at least two genes of *B. anthracis*, *ba0330* and *ba0331*, via specific differences in gene sequence, protein structure, selection pressure and available localization patterns. This study may contribute to understanding the mechanisms under which sequences evolve in their structures and how evolutionary processes enable structural variations.

## 1. Introduction

*Bacillus anthracis* and *Bacillus cereus* are Gram-positive, spore-forming bacteria that belong to the *Bacillus cereus* group, a subgroup of related Bacilli of genus *Bacillus* of the Bacillaceae family. This group (referenced as *B. cereus* sensu lato) includes *B. anthracis*, *B. cereus*, *Bacillus thuringiensis*, *Bacillus mycoides*, *Bacillus pseudomycoides* and *Bacillus weihenstephanensis* [1,2,3]. The first genomes sequenced from the *B. cereus* group that have since served as reference genomes, were that of *B. anthracis* Ames [4] and *B. cereus* strain ATCC 14579 [5], which exhibited a high degree of conserved gene order [6]. The *B. cereus* group members are phenotypically and genetically heterogeneous [7].

*Bacillus anthracis* is the etiological agent responsible for acute infectious anthrax disease in animals and its pathogenesis is primarily caused by two large plasmids. The pXO1 carries the structural genes encoding the anthrax toxin components. The protective antigen (PA), the lethal factor (LF) and the oedema factor (EF) [8] assemble into lethal toxin (LT) and oedema toxin (ET) as follows: LT (PA+LF) and ET (PA+EF). The pXO2 encodes the capsule composed of poly-γ-d-glutamic acid, in contrast to most polysaccharidic bacterial capsules including *B. cereus* [9,10,11].

*Bacillus anthracis* and *B. cereus* bacteria have a plasma membrane and thick peptidoglycan (PG) layer while they differ in the composition of the secondary cell wall components [12,13,14]. The glycan chain of PG consists of alternating units of *N*-acetylglucosamine (GlcNAc) and *N*-acetylmuramic acid (MurNAc). Enzyme activated secondary modifications of bacterial glycan strands include *N*-deacetylation, *N*-glycolylation and *O*-acetylation [15]. The *N*-deacetylation of the GlcNAc and MurNAc residues of PG correlates with the bacterium resistance of the host lysozyme, the effects on pathogen recognition via different host receptors and introduces additional positive charge into the cell wall that may increase the resistance of the bacterium to cationic antimicrobial peptides [15]. Different closely-related Bacilli strains present a wide assortment of different oligosaccharide components [16,17,18], that require different polysaccharide deacetylases (PDAs).

Polysaccharide deacetylases (PDAs) are members of the carbohydrate esterase family 4 (CE4) (CAZY database [19,20]), which catalyse the hydrolysis of either the *N*-linked acetyl group of GlcNAc and MurNAc residues (chitin deacetylases, chitooligosacharide and PG deacetylases) or *O*-linked acetyl groups from *O*-acetylxylose residues (acetylxylanesterases, xylanases) [21,22,23] through a conserved catalytic core, termed the NodB homology domain [21] (PF01522 in Pfam database [24]). Most of the CE4 members have been characterized as metalloenzymes that use acid/base catalysis with the active site metal being coordinated by a conserved catalytic Asp-His-His triad. The residues involved in the whole binding site are shared in five conserved sequence motifs (MT1-5) [25].

The genomes of *B. anthracis/B. cereus* encode for 12/11 putative or identified polysaccharide deacetylases. Initially, the biochemical characterization of two of the deacetylase gene products from *B. cereus*, Bc1960 and Bc3618, demonstrated their peptidoglycan GlcNAc deacetylase activity [26]. In *B. anthracis,* Ba1977 is also a GlcNAc peptidoglycan deacetylase involved in the resistance to the host lysozyme and is required for full virulence, whereas Ba1961 and Ba3679 participate in the biogenesis of the peptidoglycan during both elongation and cell division [27] and are involved in the anchoring of the poly-γ-d-glutamic acid capsule to the surface of *B. anthracis* [28]. In addition, Ba0424 is considered as a peptidoglycan MurNAc deacetylase [29]. Experimental data have shown that Ba0330 and Ba3679 are located around the cell envelope and septa, Ba0331 is distributed at the distinct foci, Ba1977 is located at the cell membrane and Ba2944 is located outside the cell envelope [30].

**Figure 1:**
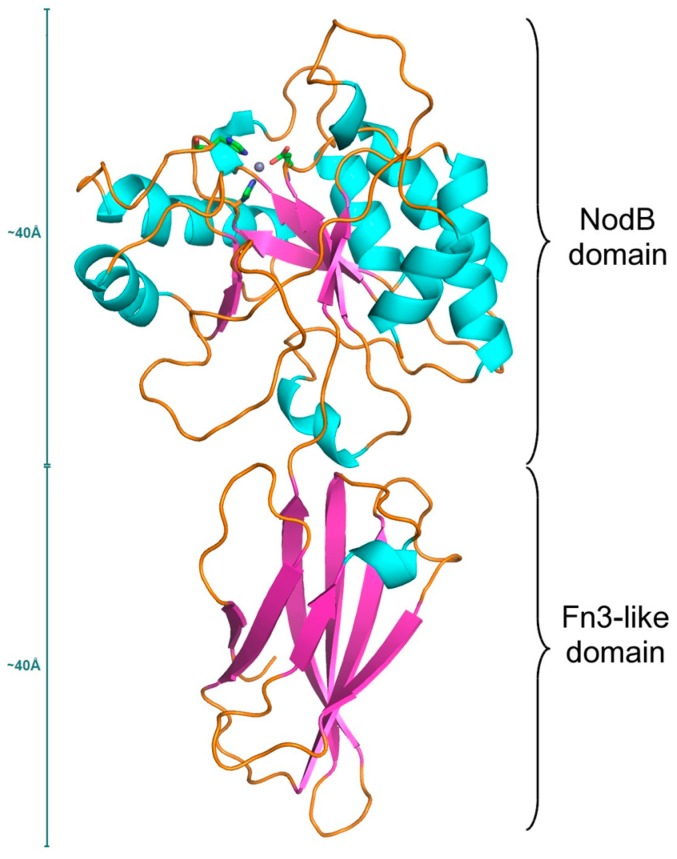
Ribbon diagram of the Ba0331 polysaccharide deacetylase (PDA) protein architecture showing the two distinct domains. Helices are represented as cyan ribbons, β-strands are in magenta and loops are shown as orange strings. The Zn ion is shown as a grey sphere with the coordinating amino acid residues (Asp213, His271 and His275) in stick representation.

The three-dimensional structures of seven *B. anthracis* and *B. cereus* PDAs are now known [29,31,32,33,34,35,36]. On the structural level, CE4 members contain at least two distinct domains. The N-terminal domain usually consists of an attachment to the membrane that contains sequences of recognition signals and a 3–5 turn α-helical segment that acts as a spacer and is believed to also act as a recognition region. Towards the C-terminal end, the catalytic NodB domain adopts an α/β barrel structure formed by 6, 7, or 8 parallel β-strands. Some CE4 members have additional N-terminal extensions with unique features for each enzyme [25,33]. *B. anthracis* Ba0331, Ba0330, Ba3943 and *B. cereus* Bc0361 and Bc3804 have these additional domains, as evidenced by their sequence. The recently determined structures of Ba0330 and Ba0331 from *B. anthracis* and also Bc0361 from *B. cereus* revealed that the extra domain adopts an Fn3-like fold (Figure 1).

Fibronectin type 3 (Fn3) domains (PF00041 in Pfam database) [24], mediate protein-protein interactions and act as ‘spacers’ for proteins to obtain the required biological function in the right place [37,38,39]. They all adopt a Greek key β-sandwich fold with three and four strands, consisting of 80–100 amino acid residues and are comprised of domain-intrinsic [40] and interaction specific regions. The former, made up of relatively conserved residues, are responsible for forming the Fn3 scaffold through a hydrogen-bond network and a hydrophobic core. Interaction specific regions are formed by exposed residues that are not always well conserved across the Fn3 family. Some surface segments or loops may form the recognition site for the Fn3 of an interacting partner protein [41,42]. Interestingly, the properties of amino acids that are presumably under weak evolutional pressure such as residues on loops or exposed on β-sheets are also preserved [43]. The scaffold is common to all Fn3 structures and endows the domain with mechanical extensibility against tension and its high refolding speed [44,45]. The interaction of the Fn3 relies on two distinct binding loops, the RGD-site in the F-G loop of Fn3 and a neighbouring exposed loop in the C’-E loop of Fn3 [45]. The RGD triplet is associated with a variety of biological processes such as cell migration and adhesion, platelet activation and signal transduction mediation [43]. As Fn3s appear sporadically in bacterial phylogenetic trees and have a high sequence similarity to those of animals, the presence of this domain in bacteria is regarded as the most convincing example of horizontal gene transfer from animal to prokaryote [46]. They are present in bacterial extracellular carbohydrases such as chitinases and amylases and PG-hydrolysing enzymes [47].

The understanding of the evolution of protein structures under functional constraint is still a subject under development but a crucial link between gene sequence and molecular and biological function [48]. In structurally ordered globular proteins, the assembled domains are characterized by a large proportion of secondary structure as well as a hydrophobic core, a mostly hydrophilic surface and for proteins with a binding function, a selectively adapted binding interface involved in protein-ligand interaction. During evolution, these regions show different rates of amino acid substitution with the hydrophobic core including residues that are the most informative for determining the topology of the native fold [48,49], evolving more slowly than the hydrophilic surface [48,50]. The binding interface is under functional constraint and evolves the slowest, with rate differences between affinity-determining and specificity-determining residues [40].

The characteristic topology of the fold maybe preserved over a relatively long evolutionary distance in protein families [48]. Selective adaptation is guided by functional and environmental constrains. When adequate genome, structural and functional information is acquired, cross correlation can lead to a better understanding of biological processes at higher levels of assembly.

Here, we performed genomics analysis by comparing all the PDA genes from the *B. anthracis* and *B. cereus* species in order to investigate their diversity and evolution within and across their PDA protein families. We used multiple phylogenetic analyses to show that while PDAs appear to be mostly vertically inherited, lateral gene transfer may have evolved from the need to address modification in different environments or different substrates, even when gene organization is highly conserved. The distinct structural domains and their amino acid sequences were aligned and used to determine evolutionary relationships among the *B. cereus* group organisms. Extensive structural comparative analysis on subgroups of PDAs enzymes from the *B. anthracis* and *B. cereus* species supports our sequence analysis conclusions and identify the shaping and interaction responsible sequence regions of the binding site that are affected by functional variation.

## 2. Materials and Methods

### 2.1. Sequence Retrieval

The protein and nucleotide sequences of the polysaccharide deacetylase gene family of *B. anthracis* str. Ames and *B. cereus* ATCC14579 were retrieved from the National Center for Biotechnology Information (NCBI, Bethesda, MD, USA) database. Details about the PDAs of the *B. anthracis* str Ames genome are listed in Table 1. Their designation, accession numbers, putative function [30], the localization prediction and the presence/absence of a cleavage site according to the LocateP database [51,52]; the Protein Data Bank [53] code—if a crystal structure exists—and their homologue from *B. cereus* ATCC14579 are included.

BLAST searches were performed using Mega BLAST [54] at the RefSeq protein database (and PDB and SwissProt) using Blastp (protein–protein BLAST) with default parameters, except for the maximum target sequences. Scores for the all BLAST alignments were in the range of *e*^−164^ to *e*^−131^ between the PDAs and their bacterial homologues.

### 2.2. Sequence Alignment

Clustal Omega, the multiple sequence alignment program (Clustal-O) [55,56], was used to perform all alignments of the gene and protein domain sequences. Default parameters were used for the alignment. DIVERGE [57,58] was employed to identify the residues that may have contributed to functional divergence between the Group 1 and Group 2 PDA enzymes as well as between the Group 2 and Group 3 clades. Type 1 functional divergence (rate shift) and Type 2 functional divergence analyses (conservation shift) were also run. The L align program [59] was used for two sequence comparison. ESPript was used to depict the sequence alignments and to incorporate the available conservation or diversity information and ENDscript was used for the 3D homology representation [60,61].

### 2.3. Phylogenetic Analysis

Trees were constructed using maximum-likelihood analysis with 500 bootstrap replications [62] for the test of phylogeny, as implemented in MEGA7 [63]. The evolutionary tree was inferred from the protein sequences using the Maximum Likelihood method, based on the Jones–Taylor–Thornton (JTT) matrix-based model [64] incorporated with the Gamma distribution model (Supplementary Table S1). The evolutionary tree was inferred from the nucleotide sequences using the Maximum Likelihood method, based on the General Time Reversible (GTR) incorporated with the Gamma distribution and invariant sites (G+I) model [65]. From the 24 models tested GTR+G+I produced the lowest BIC scores (Bayesian Information Criterion) and was considered to describe the substitution pattern for the protein-coding gene sequences (Supplementary Table S2). The tree with the highest log likelihood is shown in all phylogeny figures. Gaps/missing data were not included and uniform rates among sites were applied. Initial trees for heuristic searches were obtained automatically by applying Neighbour–Join and BioNJ algorithms to a matrix of pairwise distances, estimated using the Maximum Composite Likelihood (MCL) approach and then by selecting the topology with the superior log likelihood value.

### 2.4. Structural Analysis

All experimentally determined structures by X-ray crystallography for *B. anthracis* and *B. cereus* were included in the present study, obtained from the Protein Data Bank: Ba0424 (PDB ID: 2J13), Ba0150 (PDB ID: 4M1B), Bc0361 (PDB ID: 4HD5), Bc1960 (PDB ID: 4L1G), Ba0330 (PDB ID: 4V33), Bc1974 (PDB ID: 5N1J) and Ba0331 (PDB ID: 6GO1). Multiple structure alignments of corresponding domains (NodB, Fn3) were performed using PyMOL [66] based on RMSD optimization and sequence alignment and DALI [67], based on Dali Z-scores and structural similarity graphs [68]. ENDscript [60] has been used to depict the weak and strong sequence conservation on the structure between close members of the PDA family in order to pinpoint variable sites in the functional domain.

Additional structural models for some of the NodB domains of close homologues to the experimental structures (Ba1977, Bc2929, Ba3679, Bc0171, Bc0467 and Ba5436) were generated using homology modelling for some of the amino acid sequences of Groups 2 and 3 using as templates of the close homologues Bc1960, Bc1974, Ba0424 and Ba0150 structures with the SWISS-MODEL [69] target-template alignment automated server using default parameters or via threading with ROSETTA [70] and I-TASSER [71]. ROSETTA was run using the online server to initially find the domain homologues via the Ginzu algorithm and subsequently predict a full 3-dimensional structure using the default settings. I-TASSER was also used to evaluate the 3D models using LOMETS [72]. The structure evaluation suite of SWISS-MODEL provided measures of model quality (QMEAN) [73]; I-TASSER calculated the quality indices C-score; and TM-score and RMSD against the threading templates were used to choose the best model for the modelled sequences. The QUARK algorithm was used for the prediction of short sequences with the aid of the online server [74,75]. Template modelling on close homologues was used mainly to identify subtle changes in the binding cavity mainly due to amino acid substitutions in the studied motifs (MT1-MT5).

Structure figures were generated using PyMOL [66]. The 3V [76] web server was used for analysing all the internal volumes of the binding site.

## 3. Results

### 3.1. Phylogenetic and Sequence Analysis of the Polysaccharide Deacetylases (PDA) Gene Family

To investigate the evolutionary relationship among the *B. anthracis* and *B. cereus* PDAs and to identify additional homologue PDAs in other members of the *Bacillus cereus* group, we performed exhaustive BLAST searches through a selection of available genome and expressed sequence tags (EST) databases. To understand the phylogenetic relationship and parameters leading to the functional specialization that created the PDAs’ family of proteins, we separately analysed the sequences encoding the N-terminal (signal, localization and Fn3 when available) and the C-terminal (catalytic NodB) domains.

**Figure 2:**
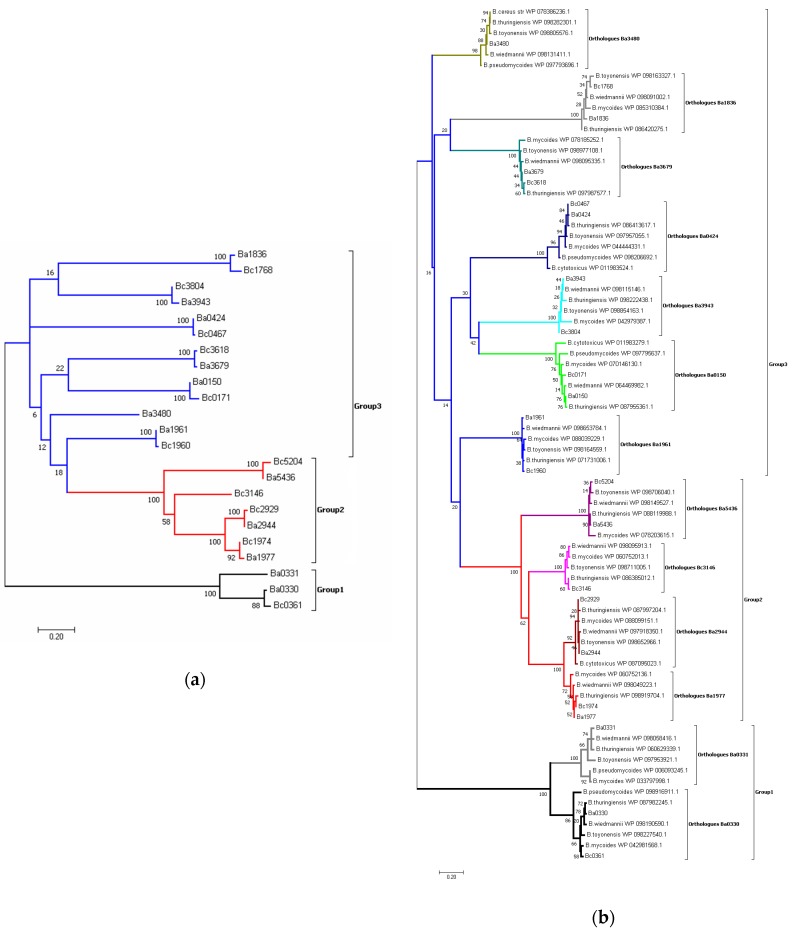
Molecular phylogenetic analysis of the PDA NodB domain. (**a**). The unrooted tree of 23 protein sequences of the PDA NodB domain from *B. anthracis* strain Ames (Ba) and *B. cereus* strain ATCC14579 (Bc) by the Maximum Likelihood method and with the highest log likelihood (−4084.19) is shown. The sequences are clustered in three groups represented with different colours (Group 1 in black, Group 2 in red and Group 3 in blue). (**b**). The unrooted tree of the NodB domain of 80 PDA amino acid sequences (homologues of the *B. anthracis* strain Ames (Ba) and *B. cereus* strain ATCC14579 (Bc) given in Figure 1a), selected from six different Bacilii species by the Maximum Likelihood method and with the highest log likelihood (−5578.53) is shown. The percentage of trees where the associated taxa clustered together is shown next to the branches. Grouping of the orthologues with respect to the *B. anthracis*/*B. cereus* species is shown in different colours.

**Figure 3:**
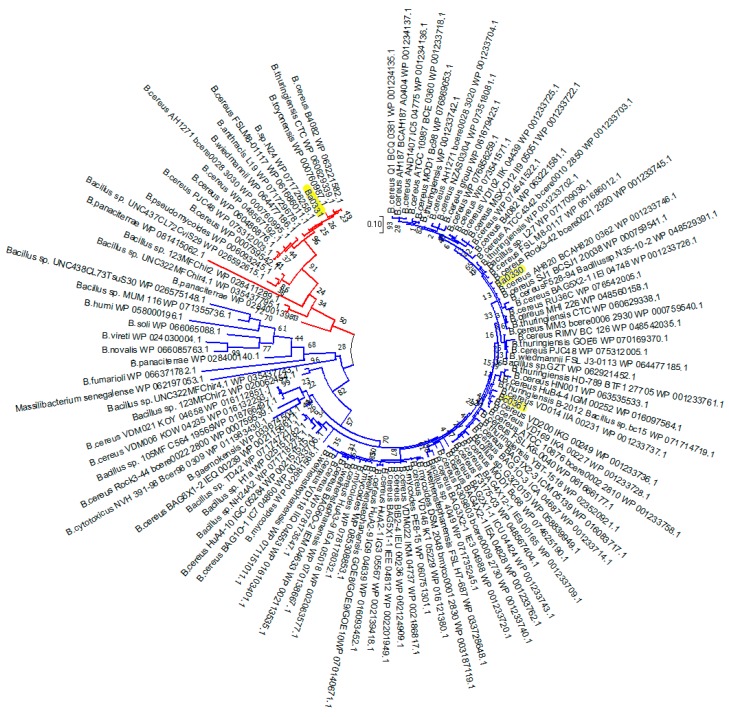
Molecular Phylogenetic analysis of the PDA Fn3 domain. The unrooted tree of 117 amino acid sequences of the PDA Fn3 domain by the Maximum Likelihood method is shown. The tree with the highest log likelihood (−10,732.65) is shown. The sequences are clustered in two distinct groups (Ba0330 like (blue) and Ba0331 like (red). Ba0330, Bc0361 and Ba0331 are highlighted in yellow. Sequences are from the Bacilii species that have been found to contain PDAs with the Fn3 domain.

Using multiple phylogenetic approaches, we consistently found that each of the NodB and Fn3 domains formed strong monophyletic groups amongst their own orthologues that were robustly supported by high bootstrap values for the NodB domains (close to 100%) and moderately high values (around 80%) for the Fn3 domain (Figure 2 and Figure 3 and Supplementary Figure S1).

For the NodB domain, 23 sequences were included in the final gene tree reconstruction. Of these, 12 sequences were from *B.anthracis* str. Ames, while 11 belonged to *B. cereus str.* ATCC14579 (Figure 2a). Extending the tree reconstruction to include further members of the Bacilii species resulted in 80 sequences of the NodB domain clustered in distinct subgroups (Figure 2b). The overall topology of the amino acid sequence tree (Figure 2a,b) corresponds closely to the nucleotide sequence tree (Supplementary Figure S1). Group 1 includes Ba0330, Bc0361 and Ba0331; Group 2 includes homologues of Bc1974, the established PG GlcNAc deacetylase; and Group 3 includes Ba0424, Ba1960 and Ba0150 and their *B.cereus* homologues. Compared in pairs, all the *B. anthracis* (Supplementary Table S3) and *B. cereus* (Supplementary Table S4) [59] PDAs had identities ranging between 21% and 43% for their NodB domain within their family. Exclusions were Ba1977-Ba2944 (75.7% identity), Bc1974-Bc2929 (76.5% identity), Bc1974-Bc3146; and Bc2929-Bc3146 (~50% identity each pair) for the NodB domain. Ba1977 was predicted to be a membrane anchored protein without a cleavage site (CS), while Ba2944 was predicted as extracellular with a cleavage site (AHTALAST) [51]. In addition, in the highly conserved proteins of Group 1, orthologous Ba0330 and Bc0361 shared a 94.5% identity (99.5% similarity) while paralogous Ba0331 and Ba0330 shared a 59.9% identity (82.4% similarity) for their NodB domain.

For the Fn3 domains found in Bacilli PDAs, 117 sequences were included in the final gene tree reconstruction after removal of the isoforms and redundant sequences. Of these, 105 sequences were from *B. cereus* group strains while 12 belonged to the genus *Bacillus*. The Fn3 domain sequences of Ba0330 and Ba0331 were clustered in two distinct groups. The separation of the Ba0330 homologues (including Bc0361) from Ba0331 and its homologues is shown in Figure 3.

In Group 1, PDAs were predicted to be N-terminally anchored to the membrane via lipid modification of the conserved cysteine residue [51,77]. On the N-terminal end, they carried a signal peptide (as predicted by the DOLOP database [78] for targeting the Sec translocation pathway (as predicted by the LocateP database) [51]. This was followed by a characteristic lipobox consensus sequence (LVI) (ASTVI) (GAS) ↓ (C) [77]. The lipoprotein precursors (Lpp) contained an N-terminal 14-amino-acid-long signal peptide, which was distinguished from the normal signal peptides by its C-terminal lipobox, comprising a conserved three-amino-acid sequence in front of an invariable cysteine. Ba0330 and its orthologue Bc0361 had LAGC and Ba0331 had LVGC. The lipobox sequence was linked to the Fn3 domain via a seven-turn α-helix for Ba0331 and a five turn helical segment for Ba0330 and Bc0361, as predicted by QUARK, acting as a spacer or hinge. Two turns of the α-helical segment containing the sequence QIQETTA were present in Ba0331 after the anchoring N-terminal segment. In addition, a 15-nucleotide insertion in the *ba0331* gene (mostly Adenines) encoded the EQKKA charged pentapeptide segment located downstream in the spacer, predicted as nearly two turns of α-helix. In total, Ba0331 was longer from Ba0330 by seven amino acids (Supplementary Figure S2). The QIQETTA α-helix forming sequence was also uniquely present in the oligomeric translocase channel formation interface of the D2 domain of the protective antigen (PA), an anthrax toxin component responsible together with the LF and EF for the virulence of *B. anthracis* (Supplementary Figure S3) [79].

**Figure 4:**
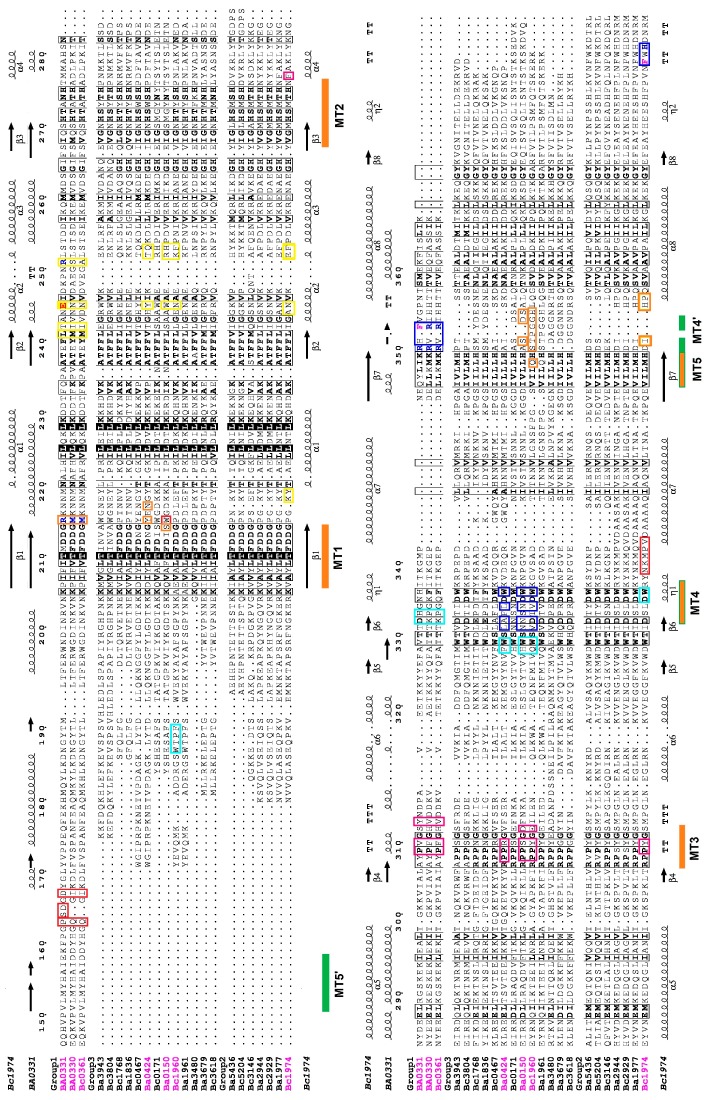
Sequence alignment diagram of the NodB domain of 23 *B. anthracis* str. Ames and *B. cereus* str. ATCC14579 PDAs. Numbering is according to Ba0331. Proteins with known tertiary structures have their name coloured purple. Highly conserved residues between the sequences are given in bold letters. Coloured bars show the conserved motifs. The amino acids found in the binding site-forming are boxed in blue, cyan, magenta, yellow, orange, or red colours, respectively. The secondary structure of Ba0331 and Bc1974 is shown on the top and bottom (α-helices as spirals, β-strands as bold arrows, turns as bold T). Diagram using the program ESPRIPT [60].

**Figure 5:**
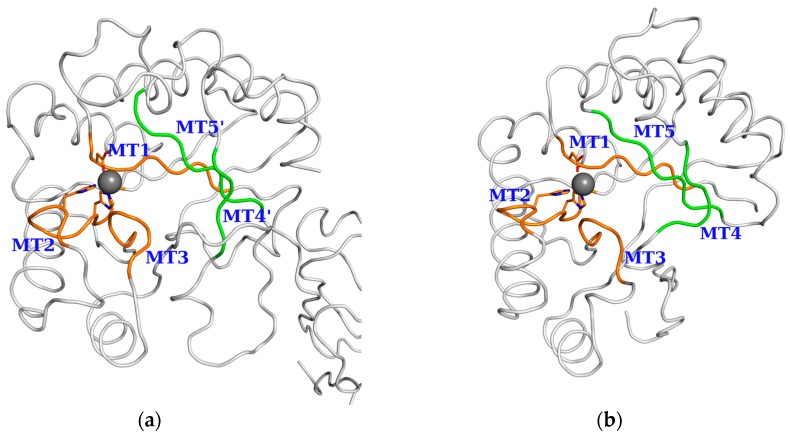
Location of conserved motifs MT1-MT5(′) on the NodB domain of Ba0331 and Bc1974 structures. The elements shown in Figure 4 sequence alignment with orange (MT1, MT2, MT3, MT4 and MT5) and green bars (MT4′ and MT5′) are shown on the 3D Cα representation of representative Group 1 and Group 2 structures. (**a**) Ba0331 and (**b**) Bc1974.

Most of the PDAs of *B. anthracis* and *B. cereus* contained the CE4 NodB catalytic domain towards the C-terminal, except for Ba3480, where the NodB domain was located between amino acid residues 295 and 491, in a total of 927. Enzyme-specific active site residues were highly conserved within the sequences of the NodB domain. They were also conserved in position in the 3D structures. In detail, three out of the five known sequence motifs associated with catalysis (MTs) [25] were conserved in all groups along the amino acid sequence. MT1 retained the sequence TFDDG, except for Ba3943 and Ba0150; MT2 with H(S/T)x(N,T,S)H, except in Ba0150; MT3 with (R/A) pPxG; and MT4 with Wxx (D,E) xxDW for Groups 2 and 3. For Group 1, MT4 became MT4′ with R (H,V) (R,F) located 15 residues further along the sequence. The sequence motif MT5 for Groups 2 and 3 was (I,V)(I,V)L(L,M,Q)H while for Group 1 proteins, the MT5′ LMYH was located in the N-terminal side of the NodB domain sequence (Figure 4 and Supplementary Figure S4). Irrespective of the shifted location of the MT4′ and MT5′ motifs in the sequence, their positions were conserved among the 3D structures of the compared enzymes (Figure 5).

### 3.2. Structural Analysis

In order to support the suggestion of differentiation in functionality and adaptation, we compared the experimental representative 3D structures available for the three sequence alignment groups. Template modelling on close homologues was also used, mainly to identify subtle changes in the binding cavity primarily due to amino acid substitutions in the studied motifs (MT1-MT5) of the NodB binding site. The significant differentiating feature among the PDA structures studied was the shape and consistence of the NodB binding site, which seemingly has adapted features that may be suitable for the accommodation of variant interacting substrates. In terms of binding site volumes, we observed variation that spanned from the rather small and deep binding site of the non-metal containing Ba0150 to the wider oligosaccharide binding site of Bc1974 or to the longer binding site of Bc1960. Even the close homologues Ba0330and Ba0331 differed substantially in that aspect, with Ba0330 having a deeper and longer binding crevice and Ba0331 being restricted in length.

An additional feature separating Group 1 PDAs from the others was the introduction of an extra domain associated with their location on the cell wall and their environment. A detailed analysis supporting these arguments follows.

#### 3.2.1. The Fn3 N-Terminal Domain

Only three (Ba0330, Ba0331 and Bc0361) of the *B. anthracis* and *B. cereus* structurally studied proteins have a similar fused architecture of two domains comprised of an N-terminal Fn3-like domain, which is not a common feature present in Bacilli and a C-terminal NodB homology domain, shared by all CE4 enzymes.

**Figure 6:**
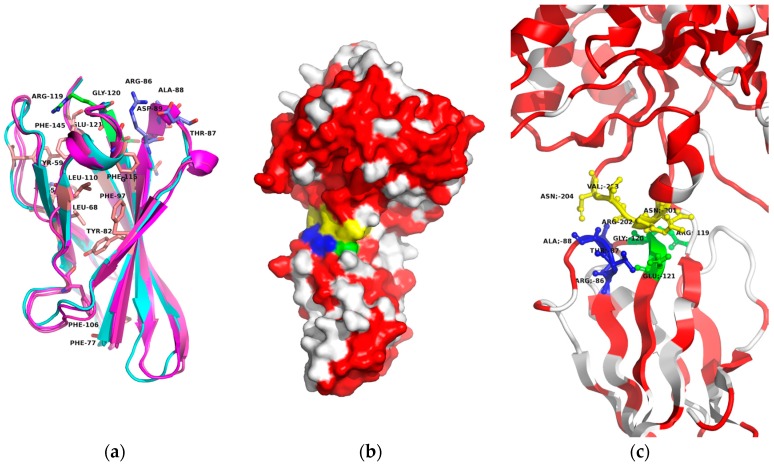
(**a**) Ribbon diagram of the superimposed Fn3 domain structures from Ba0330, Bc0361 (in shades of purple) and Ba0331 (in cyan) with the totally conserved hydrophobic residues in pink and the conserved interacting loops RTAD (res. 86–89 in blue) and RGE (res. 119–121 in green). (**b**) The Fn3 surface association with the NodB domain. (**c**) The Fn3-NodB contact in ribbon representation with amino acid residue details. Conserved loop RGE between Fn3 β5–β6 β-strands in green and loop RTAD between β3–β4 strands in blue, NodB interacting loop in yellow (res. 201–204). Conserved surface residues between Ba0330 and Ba0331 are coloured in red. Diagrams created using the program PyMOL.

Here, for the Fn3 domain, the amino acid sequence homology between Ba0330 and Bc0361 was high (84.0% sequence identity, 96.0% similarity) while between Ba0330 and Ba0331, it was distinctly lower (48.4% identity, 80% similarity) [59]. From the amino acid sequence comparison, Ba0330 and Bc0361 are orthologues between the two species, while Ba0331 is a more distant sequence. Looking at the conservation on a structural level, certain important functional features such as the beta sandwich folding motif, the RGE domain association loop and ring hydrophobic residues were conserved (Figure 6a–c). Ba0330 and Bc0361 showed a Cα RMS distance of 0.9 Å over 96 aligned residues of the Fn3 domain (50–145), while the RMS distance between Ba0330 and Ba0331 was for the same corresponding residues 1.1 Å, indicating a highly conserved fold (Supplementary Figure S5).

Two loop structures RTAD and RGE that interact directly with the NodB domain (Figure 6b,c) were totally conserved within Group 1. The latter is an integral part of Fn3 like domains with high conservation and widely discussed functionality [37,80,81]. In the Group 1 PDA case, these acted as a conserved interdomain contact area with strong electrostatic interactions towards the NodB domain (res. 201–204). Fn3 fold core forming hydrophobic residues Tyr59, Leu88, Phe77, Phe97, Phe106 and Phe115 were conserved [43] as well as Trp56 on the surface. All totally conserved residues, apart from the two interactive loops above-mentioned, were located on the inside of the beta barrel (pink thick sticks) except for Trp56. All residues on the outside of the protein domain were variable while keeping their physicochemical character (Supplementary Figure S6).

#### 3.2.2. The NodB Catalytic Domain

**Figure 7:**
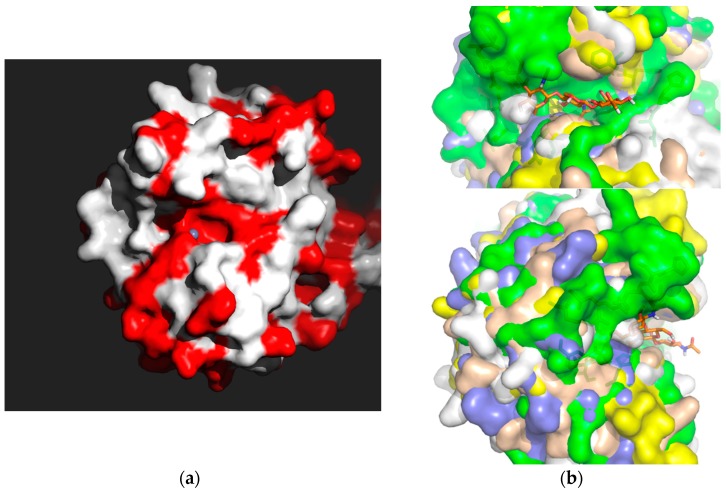
Conservation of NodB binding sites. (**a**). Surface representation of the NodB PDA binding domain facing the active site with the Group 1 amino acid residue identity (in red) for Ba0331, Ba0330 and Bc0361. The metal (Zn) ion position in the active site is indicated with a grey sphere. (**b**). Surface representation of the NodB PDA binding site from selective *B. anthracis* and *B. cereus* structurally determined NodB domains superimposed (Bc1974 (yellow), Ba0331 (blue), Ba0330 (green), Bc1960 (beige), Ba0424 (white)) in two orthogonal views to highlight the similarities (shape) and detailed differences between the PDAs. A trisaccharide GlcNAc is modelled in Bc1974 (in orange sticks) [35]. The formation of the binding crevice (running horizontally) is shown as well as the detailed differences between the PDAs on the surface.

Within the studied 23 PDAs, the general (α/β)_8_ barrel fold of the NodB domain (Figure 1) present in all CE4 esterases was conserved. Also, highly conserved was the active site containing a metal ion (shown in red around the metal ion represented with a grey sphere in Figure 7a) but missing from Ba0150, coordinated by the Asp–His–His triad at the bottom of a well-formed groove [25]. The binding cavity was elongated (Figure 7b) and some of the forming amino acid residues were clustered in five motifs (MT1-MT5) extending along the amino acid sequence, yet in spaces located on either side of the binding groove (Figure 5).

Some of these clusters were well conserved (shown with orange bars in Figure 4), while others varied in character and position across or within the three PDA groups (shown with green bars in Figure 4).

**Figure 8:**
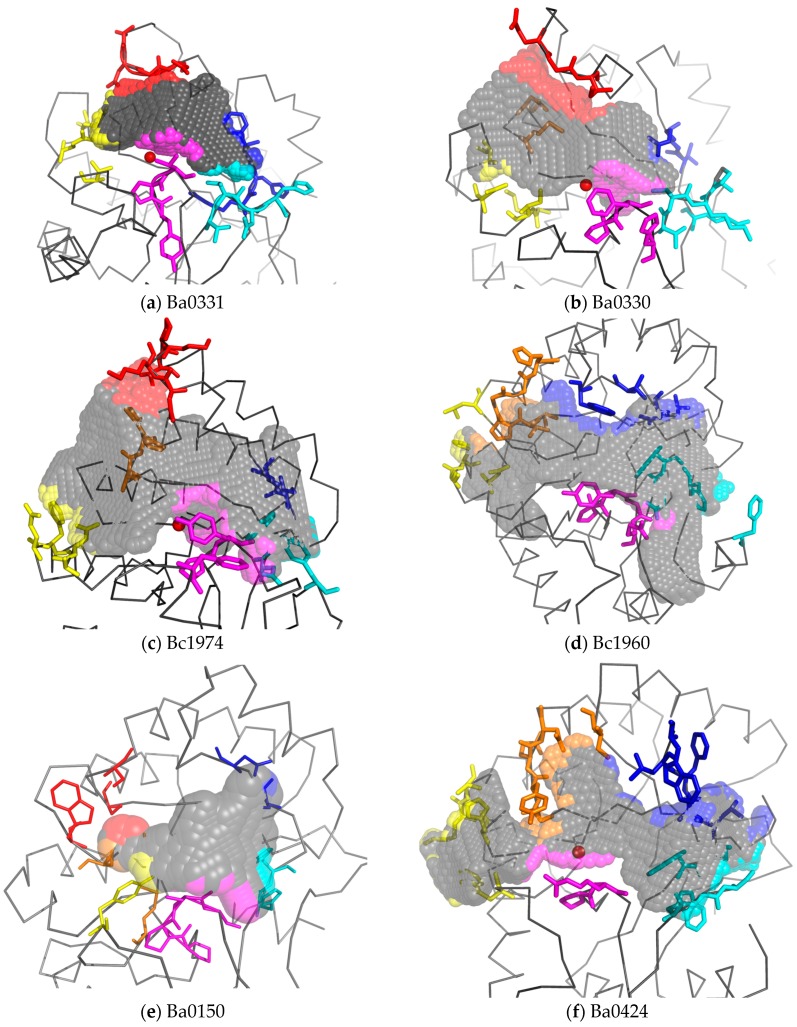
Cavity volumes of the substrate binding grooves of experimentally determined PDAs. The volume of the cavity extending on each side of the zinc ion is represented with grey spheres. The domains that set the outer boundaries of the binding groove are coloured in blue, cyan, magenta, yellow, orange, or red in accordance with Figure 4 and their corresponding amino acid residues are shown in sticks. Where applicable, the zinc ion is represented as red balls. Despite the overall similar fold of all NodB domains, the relative analysis revealed profound variation between the Fn3-containing PDAs Ba0331 (**a**) and Ba0330 (**b**) as well as between the highly homologous Bc1974 (**c**) and Bc1960 (**d**). The non-metal containing Ba0150 (**e**) has a rather small and deep crevice, while the cavity of the putative MurNAc deacetylase Ba0424 (**f**) contains a restriction ring at the post-MT1 region (coloured orange).

In detail, unlike the relatively conserved motifs MT1 and MT2, which contribute to the metal coordinating residues, the other three motifs MT3, MT4 and MT5 (Figure 4) as well other peripheral loops that frame the binding cavity and confer to the substrate specificity, presented substantial differences. The location of these motifs on the primary structure of the studied herein proteins were not highly conserved but major differences arose from the physicochemical properties of their constituent amino acids and ultimately resulted in significant variation in the binding groove volumes and shapes (Supplementary Table S5, Figure 8 and Supplementary Figure S7). Moreover, the observed variation was not subgroup specific and was present even amongst the Group 2 and Group 3 PDAs, which contained only the NodB catalytic domain (Supplementary Figure S8) or between the Group 1 Fn3-like domain containing putative PDAs. To further investigate these differences, we constructed additional structural models of the NodB domain for some of the close homologues to the experimental structures (Ba1977, Bc2929, Ba3679, Bc0171, Bc0467 and Ba5436) (Supplementary Tables S6 and S7) in Groups 2 and 3. Their comparison confirmed the overall similarity of the homologues (small all atom RMS values and positions of equivalent side chains) yet highlighted differences in the details of the binding site topology due to selected amino acid substitutions (Supplementary Figures S8 and S9).

A closer inspection of the binding grooves, illustrated in a color-coded style (Figure 8), revealed a spatial conservation of the MT3 domain (shown in magenta) close to the catalytic site in all available structures, despite the differences in their containing residues. The smallest differences were perceived amongst the spatial arrangements of the MT4 domains (shown in blue), despite the large variation in their constituent amino acids. Larger discrepancies were observed in post-MT1 and pre-MT2 loops (shown in yellow), which was also consistent with the large sequence variations. These loops set the upper boundaries of the binding groove and moreover modulated the spatial arrangement of the post-MT4 or post-MT5 loops (shown in red) either by interacting with each other when they were dominated by polar residues (forming explicit polar interactions), or by ignoring each other when they were dominated by residues of different physicochemical properties (Figure 8a,c). It should be noted that the latter loop was present only in lipid anchored PDAs and not in putative cytoplasmic ones.

**Figure 9:**
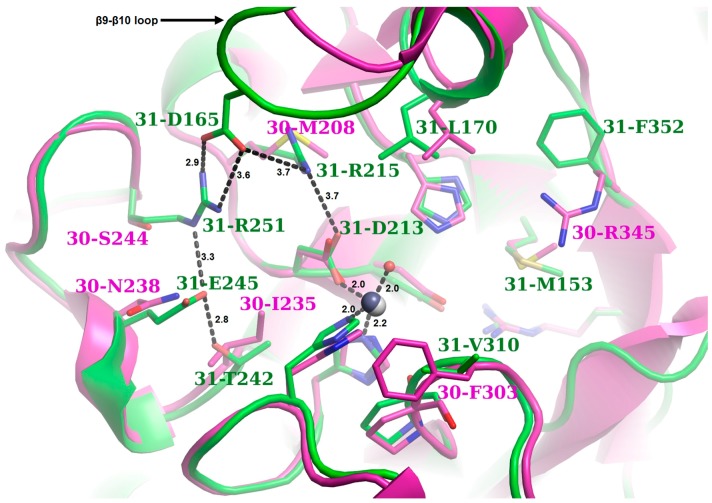
Superposition of the NodB domain containing the binding sites of Ba0331 and Ba0330. Ba0331 is shown in green and Ba0330 is shown in magenta (acquired from PDBIDs: 4V33, 6GO1). The figure is centred on the zinc ion and therefore only the binding site grooves are shown. Τhe residues that either belong directly to an MT1-5 domain or lie within a 5 Å distance from one of these motifs are represented as sticks. The acetate ions were removed for clarity reasons. A black sphere represents the zinc ion in the active site for Ba0331 and a grey sphere shows the zinc ion for Ba0330. The zinc coordination sphere or the interacting network unique in Ba0331 is shown in black dashed lines (distances in angstroms). Only the different residues between Ba0331 and Ba0330 are labelled. A strong interacting network (on the left side of the figure), which corresponds to the upper rim of the binding groove, was observed in Ba0331. In contrast to the Ba0330/Bc0361 deacetylases where the particular side is occupied by hydrophobic or small amino acids, in Ba0331, a number of charged or hydrophilic residues were located. Strong interactions of D165 with positively charged residues stabilize the β9–β10 loop to a closed conformation, thus reducing the available volume of the binding groove. On the right side of the figure, which corresponds to the lower side of the binding groove, the presence of M153, L170 and F352 characterized the boundaries as hydrophobic.

Concerning the lower rim of the binding groove (shown in cyan), which in only NodB-containing PDAs (Group 2 and Group 3) is formed by amino acid residues close to the C-terminus and in Fn3-like containing proteins (Group 1) is comprised by pre-MT4 amino acid residues, its structural location was adequately preserved. However, the participating residues varied significantly among all of the studied proteins with the exception of the Group 2 proteins (Figure 4). The MT5 domain for Group 1 proteins was located close to the N-terminus of their NodB domain (named MT5′), while the MT4 domain was shifted downwards, towards their C-terminus, by approximately 15 residues (Figure 4). Moreover, in those three proteins, the MT4 domain seemed to be electrostatically altered, bearing positively charged residue(s), contrary to other members of the family where this location is occupied by the carboxylate group of an aspartic acid (Figure 4). In particular for Ba0331, the MT4 domain presented unique features, having a mixed charged-aromatic character with the residues Arg350 and Phe352 pointing towards the binding site (Figure 9). This phenylalanine residue along with the MT5′ Met153 and Leu170 formed a hydrophobic triangle delimiting the lower part of the binding groove.

On the other side of the binding groove, Ba0331 was again an outlier of the particular area of interest. Distinctly from Ba0330 and Bc0361, charged and polar residues occupied the upper rim of the binding site. At the other two Fn3-like containing putative PDAs, this side was occupied by hydrophobic or small amino acids, whereas in Ba0331, an extensive network of salt bridges and H-bonds was observed. All these differences occurred adjacent to the zinc coordinating residues Asp213 and His275, at a maximum distance of 6 Å. Specifically, at position 215, the methionine residue found in Ba0330 and Bc0361 was replaced by an arginine, which was stabilized by interactions with Asp213 and Asp165 of the β9–β10 loop (Ile in Ba0330 and Bc0361). Moreover, Asp165 formed a strong salt bridge with Arg251 (Ser and Ala in Ba0330 and Bc0361, respectively) imposing an inward shift of the whole β9–β10 loop towards the binding cavity, which locally decreased the available volume of the binding groove for Ba0331. The β9–β10 loop in Ba0331 was extended in comparison to that of Ba0330 by the insertion of two amino acid residues (Ser164, Asp165) (Figure 9, Supplementary Figure S2). The guanidinium group of Arg251 interacted with Glu245 (Asn in Ba0330 and Bc0361) and the whole network terminated circularly with an interaction between the latter and Thr242 (Ile in Ba0330 and Bc0361).

In total, the length of the Ba0330 and Ba0331 protein molecule, considering the length of the 5-turn N-terminal α-helix (about 25 Å), the Fn3 domain (about 40 Å) and the NodB domain (about 40 Å) may reach a length of over 105 Å in total, which is a lot further from the other membrane anchored PDA enzymes such as Bc1974 and so forth.

## 4. Discussion

*Bacillus anthracis* and *B. cereus* bacilli are etiological agents of human and animal disease, either through local infection through the gastrointestinal route or by inhalation. Their disease impact has been described in ancient literature dating back more than 2000 years and still are of enormous interest in current scientific research. Cell wall-attached carbohydrate glycopolymers exhibit highly variable structures and participate in the protection, connection, or control of the major envelope constituents as well as in host-cell adhesion, inflammation and immune activation [30]. In addition, the modification of the peptidoglycan backbone is the predominant mechanism for host lysozyme resistance. The key players towards this action are peptidoglycan deacetylases. For the selected bacilli, these key enzymes for each species provide the tools for PG modification and have been the subject of intensive biochemical, structural and medicinal chemistry research.

Creation of genetic and functional flexibility is often achieved through multigene families. However, the complex processes that shape their evolution also mean that they are often composed of *mosaics* of sequences, each with a different phylogenetic history, rather than strictly homologous genes gradually diverging through time [82]. Studies have described the neofunctionalization, subfunctionalization and nonfunctionalization of proteins through the important process of gene duplication [83,84,85].

The phylogenetic analysis among the *B. anthracis* and *B. cereus* PDA NodB catalytic domain has revealed a clustering of the enzymes into three groups (1, 2 and 3), conserved along the different species. The genetic divergence of Group 1 sequences from the others was inferred from both the phylogenetic trees (Figure 2) and structure representations (Figure 8 and Figure 9). Although the same metal activating mechanism was conserved, the NodB domain was modified to accommodate different glycans. This was achieved by modifications in the binding site-forming residues clustered in sequence motifs (MTs), causing clustering of the homologue sequences on the two species evolutionary tree (Figure 1a), indicating subfunctional diversification. The same was also shown from the clustering of the homologues in ae trans-species analysis, further supporting vertical descent (Figure 2b).

Only Group 1 proteins but not Group 2 and Group 3 appeared to contain an Fn3-like domain in addition to the C-terminal catalytic domain. This implies that Group 1 PDAs may each have gained one of the original ancestral duplicate copies or have gained the Fn3 domain from horizontal evolution [43] or that Ba0330 and Ba0331 are the result of a gene duplication event. However, the duplicated paralogue of Ba0331 Fn3 containing domain was absent in most of the *B. cereus* strain genomes sampled.

Structural information provided by X-ray crystallographic studies for the NodB domain of CE4 esterases has been invaluable in understanding the biological functions of these proteins and the mechanisms by which they act on oligosaccharides and polysaccharides. Although they can be grouped based on the conservation of the sequence or protein fold, such groupings are not predictive of function. Sufficient diversity exists among the NodB catalytic domain containing family members, so functional elements, either specific amino acids or binding-site topographies, are not conserved. The differences in the 3D topography of the sequence conserved motifs forming the binding-site is a key determinant for binding specificity. Predictions of ligand specificity, based solely on the NodB fold, are inadequate. Some major factors appear to be the location of aromatic residues and loop structures that shape the binding sites to mirror the conformation of the ligand. The interaction of aromatic amino acid side chains with ligand is ubiquitous to NodB carbohydrate recognition. The side chains of tryptophan, tyrosine and, less commonly, phenylalanine form the hydrophobic platforms in NodB-binding sites, which can be long, short, or deep with respect to the active site. In addition, the size or shape of the binding site differentiated through occurring subtle structural changes may be harnessed to accommodate the conformations of the soluble oligosaccharide ligands. The amphipathic nature of carbohydrates, due to their complement of hydroxy groups, adds to their recognition through hydrogen-bond formation with polar or charged residues positioned inside or in the rim of the binding sites.

Within the polysaccharide deacetylase families present in *B. anthracis* and *B. cereus*, substrate specificity can be altered with only very few amino acid substitutions or repositioning in and around the critical pocket responsible for substrate specificity and binding (Figure 7a and Figure 9). This selectivity in specificity, due to changes in substrate-binding pocket size and shape, affects the binding or fit of the targeted substrate. However, additional variation in amino acid residues and changes in the overall structure outside the binding pocket region may have also contributed to substrate specificity differences between different enzymes. This implies that substrate specificity is also governed by amino acid sequence and structural features outside the binding region.

Albeit the many crystallographic studies of PDAs, one PDA structure with a carbohydrate bound has been resolved so far experimentally and that contains a monosaccharide (GlcNac) [86]. From the studies on the biological function of PDA family members, it is understood that the binding site of the said enzymes may accommodate different substrates and perform at different environments (location in the exo-membrane space, salinity, etc.) [30,86]. This study, following the recent crystallographic and modelling ligand binding studies on members of the family [33,34,35,36], suggests that although the catalytic domain fold and active site are well conserved, detailed changes that have been introduced within the lineages may result in the diversification of functionality.

More specifically, for the catalytic NodB domain, from the structural features forming the binding site, it was shown that three specific motifs (MT1, MT2 and MT3), containing active site involved amino acid residues, remained relatively well conserved in composition and stable in position along the sequence. Two others, MT4 and MT5, seemed to have drifted on the sequence to form a binding site suitable for the targeted modified polysaccharide or the local environment. In correlation with the early biochemical findings [30] for the binding specificity or the local environment of the PDAs in *Bacillus cereus* group species, it was inferred that within the PDA family, the genes involved have been evolved within the lineage to contribute to diversified functionality.

Some selective examples of correlation within *B. anthracis* and *B. cereus* PDA families are described here. Group 2 protein phylogeny has revealed the close relationship between Ba1977 and Ba2944 in *B. anthracis* and their orthologous counterparts in *B. cereus* (Bc1974 and Bc2929), in line with the observation that within the PDA family, proteins have the same functionality and substrate specificity in different locii [30]. This can also be inferred from the constructed model of Bc2929 (identity to Bc1974 was 76.5%), where its close fold resemblance is indicated from an RMS value of 0.6 Å to Bc1974 (Supplementary Figure S8a) for the protein backbone and a very similar binding site. In contrast, in a comparison across the Group 2 and Group 3 NodB domain structures, for Bc1974 against the Bc1960 experimental structures (identity 39.7%) and for Bc1974 against the Ba3679 model (identity to Bc1974 36%), a larger RMS fit value for the backbone (1.6 Å) was calculated and differences in the NodB binding site shape and volume were present, supporting possible substrate diversification (Supplementary Figure S9).

Collectively, the comparison of the binding grooves among the various putative PDAs revealed noticeable variability in terms of cavity size and shape as well as in terms of amino acid-type composition. Therefore, it becomes obvious that serious obstacles arise concerning the prediction of substrate specificities by similarity, or the determination of possible substrate anchoring sites or even critical residues involved in transient interactions that promote molecular recognition. It could be possible that all these termed PDA enzymes function at different levels of the bacterial cell wall biosynthetic pathways. This analysis indicates their distinct roles at a cellular level.

In a more recent PDA neofunctionalization, which may be linked to the modifications on the outside layer composition of more virulent species, sequence differences in the binding site of the closely related Ba0331 and Ba0330-Bc0361 PDAs, all containing a lipobox and the N-terminal α-helical and Fn3 domains, indicate that the need for functional diversification has led to further adaptation of the binding site on protein molecules that may exert harmonized or independent roles. Even though the sequence homology between Ba0331 and Ba0330 is high, the clustering of the homologues in the two ends of the trans-species tree indicate some evolutionary distance (Figure 2 and Figure 3). To support this, the binding cavity of the Bα0331 enzyme, compared to Ba0330, has significant differences in shape, volume, length and specific residue positions that modify the topography of the binding site, resulting in a different network and character of interactions with the substrate (Figure 8a and Figure 9, Supplementary Table S5).

In addition, for some PDAs of virulent strains, the introduction of the N-terminal helical and Fn3 additional domains that may have been adopted through gene transfer and assumed to act as spacers, or having an unknown to date mediation functionality indicated by the conserved amino acid pattern on the helix or the module-module interaction history of the Fn3 domain could affect the recognition of bacteria by host immune factors and contribute to the resistance of bacteria to host defence factors, thereby increasing pathogenicity.

## 5. Conclusions

The mechanistic understanding of the molecular functionality of the *B. anthracis* and *B. cereus* polysaccharide deacetylase families occurring through biochemical, modelling and structural analysis has enabled a protein evolutionary analysis and grouping of PDAs, both within the PDA families or across the studied species. Although protein structure is more conserved than the sequence, this study showed that small structural modifications in a multi motif assembled binding domain may cause functional diversification. This study has indicated elements of divergence in functional characteristics with concurrent conservation of distinct local structural features in the PDA enzyme family. Evolutionary functional diversification, as in the development of specificity of the PDA NodB binding domain, may have occurred through gene duplication events. Although aspects of functional diversity have been highlighted in this study, direct correlation with substrate specificity remains an important question for further structural investigation.

## Figures and Tables

**Table 1:** PDAs of the *Bacillus anthracis* str. Ames studied as well as designation, accession numbers, putative function, the localization prediction [51,52], the protein data base (PDB) ID and their homolog from *Bacillus cereus* ATCC14579.

*Bacillus anthracis* str. Ames	NCBI RefSeq: NC_003997.3 Gene ID (CDS Location)	NCBI RefSeq Protein (aa)	Possible Function	LocateP Data Base Prediction [52]: (a) by SwissProt Classification/(b) Pathway (Cleavage Site CS)	PDB ID of Corresponding X-ray Crystal Structure	Homolog *from Bacillus cereus* str. ATCC 14579. (1) Name, (2) Protein Ref. Seq (3) (aa) (4) % Identity, (5) PDB ID of X-ray Crystal Structure
BA1961	1087084, (1847060, 1847887)	NP_844369 (275)	Peptidoglycan GlcNAcdeacetylase	Cytoplasmic/Intracellular (No CS)	–	BC1960, NP_831730 (275),94.9, 4L1G
BA3679	1089257, (3383280, 3383921, complement)	NP_845942 (213)	Peptidoglycan GlcNAcdeacetylase	Cytoplasmic/Intracellular (No CS)	–	BC3618, NP_833348 (213), 97.7
BA3480	1083768, (3198882, 3201665, complement)	NP_845761 (927)	glycosyltransferase group two family protein/polysaccharide deacetylase	Membrane/multi-transmembrane (No CS)	–	–
BA1977	1086062, (1861493, 1862314)	NP_844383 (273)	Peptidoglycan GlcNAcdeacetylase	Membrane/N-terminally anchored (No CS)	–	BC1974, NP_831744 (273), 97.15 N1J
BA2944	1085595, (2708639, 2709466)	NP_845280 (275)	Peptidoglycan GlcNAcdeacetylase	Extracellular/Secretory (released) (with CS)	–	BC2929, NP_832677 (275), 94.9
BA5436	1085036, (4923836, 4924573)	NP_847604 (245)	Peptidoglycan GlcNAc deacetylase	Extracellular/Secretory (released) (with CS)	–	BC5204, NP_834868 (245), 93.5
BA0424	1087807, (444231, 445013)	NP_842967 (260)	Peptidoglycan MurNAc deacetylase	Extracellular/Secretory (released) (with CS)	2J13	BC0467, NP_830306 (260), 98.5
BA0150	1086778, (144110, 144874, complement)	NP_842717 (254)	Polysaccharide deacetylase	Membrane/N-terminally anchored (No CS)	4M1B	BC0171, NP_830050 (254), 95.3
BA1836	1086408, (1722907, 1723611, complement)	NP_844255 (234)	Polysaccharide deacetylase	Extracellular/Secretory (released) (with CS)	–	BC1768, NP_831543 (234), 91.9
BA3943	1086849, (3618407, 3619306, complement)	NP_846187 (299)	Polysaccharide deacetylase	Extracellular/Secretory (released) (with CS)	–	BC3804, NP_833526 (299), 95.7
BA0330	1085388, (338279, 339361, complement)	NP_842877 (360)	Polysaccharide deacetylase	Extracellular/Lipid anchored (with CS)	4V33	BC0361, NP_830200 (360),90.6 4HD5
BA0331	1085987, (339513, 340616, complement)	NP_842878 (367)	Polysaccharide deacetylase	Extracellular/Lipid anchored (with CS)	6GO1	_

CDS: coding sequence, CS: Cleavage Site, aa: the number of amino acid residues.

## Supplementary Materials

The following are available online at http://www.mdpi.com/2073-4425/9/8/386/s1. References [87,88] are cited in the Supplementary Materials. Figure S1: Molecular Phylogenetic analysis of the *B. anthracis* and *B. cereus* PDA NodB domain nucleotide sequences by the Maximum Likelihood method based on the General Time Reversible model. Figure S2: Sequence alignment between Ba0330 and Ba0331. Figure S3: The QIQETTA α-helix forming sequence. Figure S4: Conservation observed within Group 1 structures. Figure S5: Superposition of the Fn3 structural domains of Group 1 proteins in pairs. Figure S6: Multiple sequence alignment of the Fn3 domains from *Bacilli* PDA sequences. Figure S7: The binding sites of the experimentally determined PDAs from *B. anthracis* and *B. cereus* structures. Figure S8: Superposition of the NodB domain of the constructed model of Bc2929 on the Bc1974 structure. Figure S9: Superposition of the NodB domain of the constructed model of Ba3679 on Bc1974. Table S1: Models tested with the lowest BIC scores for PDA amino acid sequences. Table S2: Models tested with the lowest BIC scores for 23 PDA NodB domain nucleotide sequences. Table S3: Matrix of overall percent homology (identity and similarity) between the NodB domain amino acid sequences in the *B. anthracis* Ames PDA family obtained with L Align pairwise alignment program. Table S4: Matrix of overall percent homology (identity and similarity) between the NodB domain amino acid sequences in the *B. cereus* ATCC14579 PDA family obtained with L Align pairwise alignment program. Table S5: Binding cavity measurements for the selected NodB domains. Table S6: RMSD (Cα) between experimental and model structures. Table S7: Quality indices for the constructed PDA models.

## References

[1] Helgason E., Okstad O.A., Caugant D.A., Johansen H.A., Fouet A., Mock M., Hegna I., Kolsto A.B. (2000). *Bacillus anthracis*, *Bacillus cereus*, and *Bacillus thuringiensis*—One species on the basis of genetic evidence. Appl. Environ. Microbiol..

[2] Jensen G.B., Hansen B.M., Eilenberg J., Mahillon J. (2003). The hidden lifestyles of *Bacillus cereus* and relatives. Environ. Microbiol..

[3] Tourasse N.J., Helgason E., Okstad O.A., Hegna I.K., Kolsto A.-B. (2006). The *Bacillus cereus* group: Novel aspects of population structure and genome dynamics. J. Appl. Microbiol..

[4] Read T.D., Peterson S.N., Tourasse N., Baillie L.W., Paulsen I.T., Nelson K.E., Tettelin H., Fouts D.E., Eisen J.A., Gill S.R. (2003). The genome sequence of *Bacillus anthracis* Ames and comparison to closely related bacteria. Nature.

[5] Ivanova N., Sorokin A., Anderson I., Galleron N., Candelon B., Kapatral V., Bhattacharyya A., Reznik G., Mikhailova N., Lapidus A. (2003). Genome sequence of *Bacillus cereus* and comparative analysis with *Bacillus anthracis*. Nature.

[6] Økstad O.A., Kolstø A.-B., Wiedmann M., Zhang W. (2011). Genomics of Bacillus Species. Genomics of Foodborne Bacterial Pathogens.

[7] Kolsto A.-B., Tourasse N.J., Okstad O.A. (2009). What sets *Bacillus anthracis* apart from other *Bacillus* species?. Annu. Rev. Microbiol..

[8] Okinaka R.T., Cloud K., Hampton O., Hoffmaster A.R., Hill K.K., Keim P., Koehler T.M., Lamke G., Kumano S., Mahillon J. (1999). Sequence and organization of pXO1, the large *Bacillus anthracis* plasmid harboring the anthrax toxin genes. J. Bacteriol..

[9] Mock M., Fouet A. (2001). Anthrax. Annu. Rev. Microbiol..

[10] Bourgogne A., Drysdale M., Hilsenbeck S.G., Peterson S.N., Koehler T.M. (2003). Global effects of virulence gene regulators in a *Bacillus anthracis* strain with both virulence plasmids. Infect. Immun..

[11] Candela T., Fouet A. (2006). Poly-gamma-glutamate in bacteria. Mol. Microbiol..

[12] Schneewind O., Missiakas D.M. (2012). Protein secretion and surface display in Gram-positive bacteria. Philos. Trans. R. Soc. Lond. B Biol. Sci..

[13] Fouet A., Mesnage S. (2002). *Bacillus anthracis* cell envelope components. Curr. Top. Microbiol. Immunol..

[14] Zipperle G.F.J., Ezzell J.W.J., Doyle R.J. (1984). Glucosamine substitution and muramidase susceptibility in *Bacillus anthracis*. Can. J. Microbiol..

[15] Vollmer W. (2008). Structural variation in the glycan strands of bacterial peptidoglycan. FEMS Microbiol. Rev..

[16] Fouet A. (2009). The surface of *Bacillus anthracis*. Mol. Asp. Med..

[17] Missiakas D., Schneewind O. (2017). Assembly and Function of the *Bacillus anthracis* S-Layer. Annu. Rev. Microbiol..

[18] Choudhury B., Leoff C., Saile E., Wilkins P., Quinn C.P., Kannenberg E.L., Carlson R.W. (2006). The structure of the major cell wall polysaccharide of *Bacillus anthracis* is species-specific. J. Biol. Chem..

[19] Lombard V., GolacondaRamulu H., Drula E., Coutinho P.M., Henrissat B. (2014). The carbohydrate-active enzymes database (CAZy) in 2013. Nucleic Acids Res..

[20] CAZY Database. http://www.cazy.org.

[21] Caufrier F., Martinou A., Dupont C., Bouriotis V. (2003). Carbohydrate esterase family 4 enzymes: Substrate specificity. Carbohydr. Res..

[22] Tsigos I., Martinou A., Kafetzopoulos D., Bouriotis V. (2000). Chitin deacetylases: New, versatile tools in biotechnology. Trends Biotechnol..

[23] Kafetzopoulos D., Martinou A., Bouriotis V. (1993). Bioconversion of chitin to chitosan: Purification and characterization of chitin deacetylase from *Mucorrouxii*. Proc. Natl. Acad. Sci. USA.

[24] Pfam Database. https://pfam.xfam.org.

[25] Blair D.E., Schuttelkopf A.W., MacRae J.I., van Aalten D.M.F. (2005). Structure and metal-dependent mechanism of peptidoglycan deacetylase, a streptococcal virulence factor. Proc. Natl. Acad. Sci. USA.

[26] Psylinakis E., Boneca I.G., Mavromatis K., Deli A., Hayhurst E., Foster S.J., Varum K.M., Bouriotis V. (2005). Peptidoglycan *N*-acetylglucosamine deacetylases from *Bacillus cereus*, highly conserved proteins in *Bacillus anthracis*. J. Biol. Chem..

[27] Balomenou S., Fouet A., Tzanodaskalaki M., Couture-Tosi E., Bouriotis V., Boneca I.G. (2013). Distinct functions of polysaccharide deacetylases in cell shape, neutral polysaccharide synthesis and virulence of *Bacillus anthracis*. Mol. Microbiol..

[28] Candela T., Balomenou S., Aucher W., Bouriotis V., Simore J.-P., Fouet A., Boneca I.G. (2014). N-acetylglucosamine deacetylases modulate the anchoring of the gamma-glutamyl capsule to the cell wall of *Bacillus anthracis*. Microb. Drug Resist..

[29] Oberbarnscheidt L., Taylor E.J., Davies G.J., Gloster T.M. (2007). Structure of a carbohydrate esterase from *Bacillus anthracis*. Proteins.

[30] Balomenou S., Arnaouteli S., Koutsioulis D., Fadouloglou V., Bouriotis V., Atta-ur-Rahman, IgbalChoudharyeds M. (2015). Polysaccharide Deacetylases: New Antibacterial Drug Targets. Frontiers in Anti-Infective Drug Discovery.

[31] Fadouloglou V.E., Kapanidou M., Agiomirgianaki A., Arnaouteli S., Bouriotis V., Glykos N.M., Kokkinidis M. (2013). Structure determination through homology modelling and torsion-angle simulated annealing: Application to a polysaccharide deacetylase from *Bacillus cereus*. Acta Crystallogr. D Biol. Crystallogr..

[32] Strunk R.J., Piemonte K.M., Petersen N.M., Koutsioulis D., Bouriotis V., Perry K., Cole K.E. (2014). Structure determination of BA0150, a putative polysaccharide deacetylase from *Bacillus anthracis*. Acta Crystallogr. Sect. F Struct. Biol. Commun..

[33] Arnaouteli S., Giastas P., Andreou A., Tzanodaskalaki M., Aldridge C., Tzartos S.J., Vollmer W., Eliopoulos E., Bouriotis V. (2015). Two Putative Polysaccharide Deacetylases Are Required for Osmotic Stability and Cell Shape Maintenance in *Bacillus anthracis*. J. Biol. Chem..

[34] Fadouloglou V.E., Balomenou S., Aivaliotis M., Kotsifaki D., Arnaouteli S., Tomatsidou A., Efstathiou G., Kountourakis N., Miliara S., Griniezaki M. (2017). Unusual alpha-Carbon Hydroxylation of Proline Promotes Active-Site Maturation. J. Am. Chem. Soc..

[35] Giastas P., Andreou A., Papakyriakou A., Koutsioulis D., Balomenou S., Tzartos S.J., Bouriotis V., Eliopoulos E.E. (2018). Structures of the Peptidoglycan N-Acetylglucosamine Deacetylase Bc1974 and Its Complexes with Zinc Metalloenzyme Inhibitors. Biochemistry.

[36] Andreou A., Giastas P., Arnaoutely S., Tzanodaskalaki M., Tzartos S.J., Bethanis K., Bouriotis V., Eliopoulos E.E. (2018). Cloning, expression, crystallization, and structure determination of putative polysaccharide deacetylase Ba0331 *Acta Crystallogr*. Sect. F Struct. Biol. Commun..

[37] Hynes R.O., Rich A. (1990). Structure of fibronectins. Fibronectins.

[38] Strater N., Klabunde T., Tucker P., Witzel H., Krebs B. (1995). Crystal structure of a purple acid phosphatase containing a dinuclear Fe(III)-Zn(II) active site. Science.

[39] Sharma A., Askari J.A., Humphries M.J., Jones E.Y., Stuart D.I. (1999). Crystal structure of a heparin- and integrin-binding segment of human fibronectin. EMBO J..

[40] Bateman A., Jouet M., MacFarlane J., Du J.S., Kenwrick S., Chothia C. (1996). Outline structure of the human L1 cell adhesion molecule and the sites where mutations cause neurological disorders. EMBO J..

[41] Leahy D.J., Aukhil I., Erickson H.P. (1996). 2.0 A crystal structure of a four-domain segment of human fibronectin encompassing the RGD loop and synergy region. Cell.

[42] Main A.L., Harvey T.S., Baron M., Boyd J., Campbell I.D. (1992). The three-dimensional structure of the tenth type III module of fibronectin: An insight into RGD-mediated interactions. Cell.

[43] Jee J.-G., Ikegami T., Hashimoto M., Kawabata T., Ikeguchi M., Watanabe T., Shirakawa M. (2002). Solution structure of the fibronectin type III domain from *Bacillus circulans*. J. Biol. Chem..

[44] Oberhauser A.F., Marszalek P.E., Erickson H.P., Fernandez J.M. (1998). The molecular elasticity of the extracellular matrix protein tenascin. Nature.

[45] Plaxco K.W., Spitzfaden C., Campbell I.D., Dobson C.M. (1996). Rapid refolding of a proline-rich all-beta-sheet fibronectin type III module. Proc. Natl. Acad. Sci. USA.

[46] Bork P., Doolittle R.F. (1992). Proposed acquisition of an animal protein domain by bacteria. Proc. Natl. Acad. Sci. USA.

[47] Little E., Bork P., Doolittle R.F. (1994). Tracing the spread of fibronectin type III domains in bacterial glycohydrolases. J. Mol. Evol..

[48] Siltberg-Liberles J., Grahnen J.A., Liberles D.A. (2011). The Evolution of Protein Structures and Structural Ensembles under Functional Constraint. Genes.

[49] Pereira de Araujo A.F., Onuchic J.N. (2009). A sequence-compatible amount of native burial information is sufficient for determining the structure of small globular proteins. Proc. Natl. Acad. Sci. USA.

[50] Roth C., Liberles D.A. (2006). A systematic search for positive selection in higher plants (Embryophytes). BMC Plant Biol..

[51] Zhou M., Boekhorst J., Francke C., Siezen R.J. (2008). LocateP: Genome-scale subcellular-location predictor for bacterial proteins. BMC Bioinform..

[52] LocateP DataBase. http://www.cmbi.ru.nl/locatep-db/cgi-bin/locatepdb.py.

[53] Berman H.M., Westbrook J., Feng Z., Gilliland G., Bhat T.N., Weissig H., Shindyalov I.N., Bourne P.E. (2000). The Protein Data Bank. Nucleic Acids Res..

[54] Basic Local Alignment Search Tool. http://www.ncbi.nlm.nih.gov/BLAST/.

[55] Sievers F., Wilm A., Dineen D., Gibson T.J., Karplus K., Li W., Lopez R., McWilliam H., Remmert M., Soding J. (2011). Fast, scalable generation of high-quality protein multiple sequence alignments using Clustal Omega. Mol. Syst. Biol..

[56] Clustal Omega. https://www.ebi.ac.uk/Tools/msa/clustalo/.

[57] Gu X., Vander Velden K. (2002). DIVERGE: Phylogeny-based analysis for functional-structural divergence of a protein family. Bioinformatics.

[58] Gu X., Zou Y., Su Z., Huang W., Zhou Z., Arendsee Z., Zeng Y. (2013). An update of DIVERGE software for functional divergence analysis of protein family. Mol. Biol. Evol..

[59] Huang X., Miller W. (1991). A time-efficient, linear-space local similarity algorithm. Adv. Appl. Math..

[60] Gouet P., Robert X., Courcelle E. (2003). ESPript/ENDscript: Extracting and rendering sequence and 3D information from atomic structures of proteins. Nucleic Acids Res..

[61] Robert X., Gouet P. (2014). Deciphering key features in protein structures with the new ENDscript server. Nucleic Acids Res..

[62] Felsenstein J. (1985). Confidence limits on phylogenies: An approach using the bootstrap. Evolution.

[63] Kumar S., Stecher G., Tamura K. (2016). MEGA7: Molecular Evolutionary Genetics Analysis Version 7.0 for Bigger Datasets. Mol. Biol. Evol..

[64] Jones D.T., Taylor W.R., Thornton J.M. (1992). The rapid generation of mutation data matrices from protein sequences. Comput. Appl. Biosci. CABIOS.

[65] Nei M., Kumar S. (2000). Molecular Evolution and Phylogenetics.

[66] (2015). The PyMOL Molecular Graphics System.

[67] Holm L., Laakso L.M. (2016). Dali server update. Nucleic Acids Res..

[68] Holm L., Kaariainen S., Rosenstrom P., Schenkel A. (2008). Searching protein structure databases with DaliLite v.3. Bioinformatics.

[69] Waterhouse A., Bertoni M., Bienert S., Studer G., Tauriello G., Gumienny R., Heer F.T., de Beer T.A.P., Rempfer C., Bordoli L. (2018). SWISS-MODEL: Homology modelling of protein structures and complexes. Nucleic Acids Res..

[70] Kim D.E., Chivian D., Baker D. (2004). Protein structure prediction and analysis using the Robetta server. Nucleic Acids Res..

[71] Yang J., Zhang Y. (2015). Protein Structure and Function Prediction Using I-TASSER. Curr. Protoc. Bioinform..

[72] Wu S., Zhang Y. (2007). LOMETS: A local meta-threading-server for protein structure prediction. Nucleic Acids Res..

[73] Benkert P., Tosatto S.C.E., Schomburg D. (2008). QMEAN: A comprehensive scoring function for model quality assessment. Proteins.

[74] Xu D., Zhang Y. (2012). Ab initio protein structure assembly using continuous structure fragments and optimized knowledge-based force field. Proteins.

[75] Xu D., Zhang Y. (2011). Improving the Physical Realism and Structural Accuracy of Protein Models by a Two-Step Atomic-Level Energy Minimization. Biophys. J..

[76] Voss N.R., Gerstein M. (2010). 3V: Cavity, channel and cleft volume calculator and extractor. Nucleic Acids Res..

[77] DOLOP Database. www.mrc-lmb.cam.ac.uk/genomes/dolop/table.shtml.

[78] Babu M.M., Priya M.L., Selvan A.T., Madera M., Gough J., Aravind L., Sankaran K. (2006). A database of bacterial lipoproteins (DOLOP) with functional assignments to predicted lipoproteins. J. Bacteriol..

[79] Feld G.K., Thoren K.L., Kintzer A.F., Sterling H.J., Tang I.I., Greenberg S.G., Williams E.R., Krantz B.A. (2010). Structural basis for the unfolding of anthrax lethal factor by protective antigen oligomers. Nat. Struct. Mol. Biol..

[80] Spitzfaden C., Grant R., Mardon H., Campbell I. (1997). Module-module interactions in the cell binding region of fibronectin: Stability, flexibility and specificity. J. Mol. Biol..

[81] Potts J.R., Campbell I.D. (1994). Fibronectin structure and assembly. Curr. Opin. Cell Biol..

[82] Page D.M.R., Holmes C.E. (2009). Molecular Evolution, A Phylogenetic Approach.

[83] Konrad A., Teufel A.I., Grahnen J.A., Liberles D.A. (2011). Toward a general model for the evolutionary dynamics of gene duplicates. Genome Biol. Evol..

[84] Hughes A.L. (1994). The evolution of functionally novel proteins after gene duplication. Proc. Biol. Sci..

[85] Rastogi S., Liberles D.A. (2005). Subfunctionalization of duplicated genes as a transition state to neofunctionalization. BMC Evol. Biol..

[86] Blair D. E., van Aalten D.M.F. (2004). Structures of *Bacillus subtilis* PdaA, a family 4 carbohydrate esterase, and a complex with N-acetyl-glucosamine. FEBS Lett..

[87] Zhang Y., Skolnick J. (2004). Scoring function for automated assessment of protein structure template quality. Proteins.

[88] Kintzer A.F., Thoren K.L., Sterling H.J., Dong K.C., Feld G.K., Tang I.I., Zhang T.T., Williams E.R., Berger J.M., Krantz B.A. (2009). The protective antigen component of anthrax toxin forms functional octameric complexes. J. Mol. Biol..

