# Supplementary material for: Structural and Evolutionary Insights within the Polysaccharide Deacetylase Gene Family of Bacillus anthracis and Bacillus cereus
Source: Genes (Basel). 2018 Jul 31;9(8):386. doi: 10.3390/genes9080386 (PMC6115787; doi:10.3390/genes9080386)
Supplement: Supplementary file 1 [file genes-09-00386-s001.pdf]

genes

MDPI

Supplementary Data

Structural and Evolutionary Insights within the Polysaccharide Deacetylase Gene Family of *Bacillus anthracis* and *Bacillus cereus*.

Athena Andreou[1], Petros Giastas[2,‡], Elias Christoforides[1]and Elias E. Eliopoulos[1,*]

[1] Department of Biotechnology, Agricultural University of Athens, Iera Odos 75, 11855 Athens, Greece;
[2] Department of Neurobiology, Hellenic Pasteur Institute, Vasilissis Sofias 127, 11521 Athens, Athens, Greece
‡ Current address: INRASTES, National Centre for Scientific Research Demokritos, 15341, Athens, Greece

* Correspondence: eliop@aua.gr; Tel.: +30-210-529-4223

Table S1: Models tested with the lowest BIC scores for 148 PDA amino acid sequences.

Model	#Param	BIC	AICc	lnL	Invariant
JTT+G	294	11326.52	9128.95	-4263.97	n/a
JTT+G+I	295	11335.67	9130.68	-4263.78	0.01
WAG+G	294	11436.68	9239.11	-4319.04	n/a
WAG+G+I	295	11445.60	9240.61	-4318.75	0.01
LG+G	294	11451.77	9254.21	-4326.59	n/a
JTT+I	294	11453.87	9256.31	-4327.64	0.05
LG+G+I	295	11459.63	9254.63	-4325.76	0.03
Dayhoff+G	294	11474.05	9276.49	-4337.73	n/a
JTT	293	11480.46	9290.33	-4345.70	n/a
Dayhoff+G+I	295	11483.57	9278.58	-4337.73	0.00
mtREV24+G	294	11506.77	9309.20	-4354.09	n/a
mtREV24+G+I	295	11514.86	9309.87	-4353.38	0.02
rtREV+G	294	11540.33	9342.76	-4370.87	n/a
rtREV+G+I	295	11547.11	9342.11	-4369.50	0.03
JTT+G+F	313	11549.05	9210.38	-4284.80	n/a
JTT+G+I+F	314	11558.79	9212.70	-4284.91	0.00
WAG+I	294	11559.48	9361.92	-4380.45	0.05
WAG	293	11582.98	9392.84	-4396.96	n/a
cpREV+G	294	11586.71	9389.15	-4394.06	n/a
LG+I	294	11588.91	9391.35	-4395.16	0.05
cpREV+G+I	295	11609.01	9404.01	-4400.45	0.00
Dayhoff+I	294	11611.30	9413.74	-4406.36	0.05
LG	293	11613.29	9423.15	-4412.11	n/a
Dayhoff	293	11638.23	9448.09	-4424.58	n/a
rtREV+I	294	11670.81	9473.24	-4436.11	0.05

LG+G+F	313	11681.96	9343.29	-4351.26	n/a
JTT+I+F	313	11682.45	9343.78	-4351.50	0.05
JTT+F	312	11687.76	9356.51	-4358.92	n/a
LG+G+I+F	314	11691.29	9345.20	-4351.16	0.01
mtREV24+I	294	11694.15	9496.58	-4447.78	0.05
rtREV	293	11695.77	9505.64	-4453.35	n/a
WAG+G+F	313	11704.33	9365.66	-4362.44	n/a
mtREV24+G+F	313	11710.87	9372.20	-4365.71	n/a
WAG+G+I+F	314	11713.85	9367.76	-4362.44	0.00
Dayhoff+G+F	313	11716.12	9377.45	-4368.34	n/a
mtREV24+G+I+F	314	11720.39	9374.29	-4365.71	0.00
Dayhoff+G+I+F	314	11725.64	9379.55	-4368.34	0.00
mtREV24	293	11727.12	9536.98	-4469.02	n/a
cpREV	293	11736.73	9546.60	-4473.83	n/a
cpREV+I	294	11773.95	9576.39	-4487.68	0.03
rtREV+G+F	313	11786.43	9447.76	-4403.49	n/a
rtREV+G+I+F	314	11795.14	9449.05	-4403.09	0.02
LG+I+F	313	11812.25	9473.58	-4416.40	0.05
LG+F	312	11824.47	9493.23	-4427.27	n/a
WAG+I+F	313	11833.62	9494.95	-4427.09	0.05
mtREV24+F	312	11834.37	9503.12	-4432.22	n/a
mtREV24+I+F	313	11834.45	9495.78	-4427.50	0.04
WAG+F	312	11840.38	9509.14	-4435.23	n/a
Dayhoff+I+F	313	11867.57	9528.90	-4444.06	0.05
Dayhoff+F	312	11895.13	9563.88	-4462.60	n/a
rtREV+I+F	313	11915.62	9576.95	-4468.09	0.05
rtREV+F	312	11933.40	9602.16	-4481.74	n/a
cpREV+G+F	313	11939.93	9601.26	-4480.24	n/a
cpREV+G+I+F	314	11951.98	9605.89	-4481.51	0.00
cpREV+F	312	12113.91	9782.67	-4571.99	n/a
cpREV+I+F	313	12150.27	9811.60	-4585.41	0.02

Models with the lowest BIC scores (Bayesian Information Criterion) are considered to describe the substitution pattern. For each model, AIC c value (Akaike Information Criterion, corrected), Maximum Likelihood value (lnL), and the number of parameters (including branch lengths) are also presented. Non-uniformity of evolutionary rates among sites may be modeled by using a discrete Gamma distribution (+G) with 5 rate categories and by assuming that a certain fraction of sites are evolutionarily invariable (+I). Whenever applicable, estimates of gamma shape parameter and/or the estimated fraction of invariant sites are shown. The analysis involved 148 amino acid sequences. All positions containing gaps and missing data were eliminated. There were a total of 92 positions in the final dataset. Evolutionary analyses were conducted in MEGA7. Abbreviations: GTR: General Time Reversible; JTT: Jones-Taylor-Thornton; rtREV: General Reverse Transcriptase; cpREV: General Reversible Chloroplast; mtREV24: General Reversible Mitochondrial.

Table S2: Models tested with the lowest BIC scores for 23 PDA NodB domain nucleotide sequences.

Model	#Param	BIC	AICc	lnL
GTR+G+I	53	13688.32	13312.57	-6603.0
HKY+G+I	49	13703.56	13356.13	-6628.8
T92+G+I	47	13703.69	13370.42	-6638.0
TN93+G+I	50	13709.02	13354.51	-6627.0
GTR+G	52	13751.45	13382.78	-6639.1
T92+G	46	13753.39	13427.20	-6667.4
HKY+G	48	13756.39	13416.04	-6659.8
TN93+G	49	13762.58	13415.15	-6658.3
K2+G	45	13946.73	13627.62	-6768.6
T92+I	46	13950.28	13624.09	-6765.8
K2+G+I	46	13955.09	13628.90	-6768.2
HKY+I	48	13958.61	13618.26	-6760.9
TN93+I	49	13963.93	13616.50	-6759.0
GTR+I	52	13967.55	13598.88	-6747.1
T92	45	14036.75	13717.64	-6813.6
HKY	47	14048.90	13715.63	-6810.6
TN93	48	14057.73	13717.37	-6810.4
GTR	51	14059.29	13697.70	-6797.6
JC+G	44	14077.44	13765.41	-6838.5
JC+G+I	45	14084.32	13765.21	-6837.4
K2+I	45	14115.23	13796.12	-6852.8
K2	44	14197.12	13885.09	-6898.3
JC+I	44	14207.70	13895.67	-6903.6
JC	43	14286.21	13981.26	-6947.4

Models with the lowest BIC scores (Bayesian Information Criterion) are considered to describe the substitution pattern. For each model, AICc value (Akaike Information Criterion, corrected), Maximum Likelihood value (lnL), and the number of parameters (including branch lengths) are presented. Non-uniformity of evolutionary rates among sites may be modeled by using a discrete Gamma distribution (+G) with 5 rate categories and by assuming that a certain fraction of sites are evolutionarily invariable (+I). The analysis involved 23 nucleotide sequences. Codon positions included were 1st+2nd+3rd+Noncoding. All positions containing gaps and missing data were eliminated. There were a total of 390 positions in the final dataset. Evolutionary analyses were conducted in MEGA7.

Table S3: Matrix of overall percent homology (identity and similarity) between the NodB domain amino acid sequences in the *B. anthracis* Ames PDA family obtained with L Align pairwise alignment program.

NodB	Ba0331	Ba0330	Ba3679	Ba3943	Ba0424	Ba1961	Ba1836	Ba3480	Ba0150	Ba1977	Ba2944	Ba5436
**Ba0331**		59.9	29.2	28.3	27.9	27.8	25.6	24.3	24.0	24.6	23.1	21.0
**Ba0330**	82.4		25.4	25.9	29.5	23.4	30.6	23.9	30.3	30.6	27.9	23.4
**Ba3679**	54.0	57.7		28.4	33.0	37.4	32.8	38.9	36.7	35.0	32.3	29.9
**Ba3943**	59.6	54.5	67.5		31.1	31.2	27.1	31.0	30.2	28.9	27.4	28.4
**Ba0424**	61.2	62.0	64.5	69.4		33.8	27.7	33.2	30.8	29.9	27.7	30.1
**Ba1961**	58.3	63.9	71.3	67.3	65.7		32.5	39.6	29.0	40.2	33.0	28.1
**Ba1836**	58.3	64.9	68.2	64.1	62.4	63.4		30.3	25.4	31.8	29.1	28.1
**Ba3480**	59.1	61.5	73.2	65.0	65.3	71.1	63.6		32.8	34.7	34.0	33.8
**Ba0150**	55.8	69.7	68.8	70.4	68.2	68.2	65.8	67.2		33.3	31.5	27.4
**Ba1977**	51.6	57.7	65.5	60.2	61.3	68.8	60.6	60.2	64.1		75.7	40.9
**Ba2944**	56.2	59.5	62.6	58.5	57.7	63.8	60.7	60.0	59.4	91.7		39.8
**Ba5436**	54.3	52.1	59.7	58.9	56.6	65.9	65.9	62.6	61.1	72.6	69.7	

Identity % (upper triangle)

Similarity % (lower triangle)

Table S4: Matrix of overall percent homology (identity and similarity) between the NodB domain amino acid sequences in the *B. cereus* ATCC 14579 PDA family obtained with L Align pairwise alignment program.

**NodB**	**Bc0361**	**Bc3618**	**Bc3804**	**Bc0467**	**Bc1960**	**Bc1768**	**Bc3146**	**Bc0171**	**Bc1974**	**Bc2929**	**Bc5204**
**Bc0361**		26.1	27.7	27.9	24.8	31.0	23.8	25.8	28.8	27.6	22.5
**Bc3618**	58.5		28.4	33.0	36.4	32.8	29.2	36.2	34.0	31.8	31.1
**Bc3804**	56.2	67.5		32.1	31.2	26.0	29.9	29.6	28.9	27.8	29.5
**Bc0467**	61.2	64.5	69.4		32.9	27.0	31.8	31.7	29.4	29.1	30.6
**Bc1960**	62.1	71.3	68.3	65.7		32.8	35.2	29.0	39.7	35.9	28.6
**Bc1768**	62.1	68.7	65.6	61.9	65.1		30.6	23.3	29.8	30.6	29.6
**Bc3146**	55.8	59.0	58.8	54.7	64.2	60.7		28.6	50.0	51.1	41.8
**Bc0171**	67.7	68.8	70.9	67.8	68.2	64.2	58.2		32.3	31.8	26.1
**Bc1974**	55.0	65.0	60.7	60.8	68.3	59.1	75.7	63.1		76.5	39.9
**Bc2929**	60.0	61.1	59.0	58.6	64.1	60.7	76.2	57.5	91.3		43.1
**Bc5204**	51.4	63.2	61.1	56.0	64.9	61.6	76.0	60.1	71.2	70.8	

Identity %

Similarity %

Table S5: Binding cavity measurements for the selected NodB domains.

NodB binding cavity	Volume (Å^3)	Length (Å)	Width (Å)
Ba0331	1577	27	11
Ba0330	2731	33	11
Bc1974	4528	35	11
Bc1960	4206	24	8.5
Ba0424	3728	21	11
Ba0150	1097	15	11

Table S6: RMSD (Cα) between experimental and model structures. **RMSD** is an average distance of all residue pairs (Ca) in two structures.

Model	Structure xray-PDB code	Group2	Group3			Group1		
		Bc1974-5N1J	Bc1960-4L1G	Ba0150-4M1B	Ba0424-2J13	Ba0330-4V33	Bc0361-4HD5	Ba0331-6GO1
Group2	Ba1977	0.4	2.3	2.1	2.4	2.8	2.8	3.8
	Bc2929	0.6	1.8	2.0	2.2	2.5	2.5	2.4
	Ba5436	0.6	2.0	2.0	2.5	2.3	2.2	2.1
Group3	Ba3679	1.6	0.9	0.9	1.5	2.3	2.5	2.4
	Bc0171	2.3	1.1	0.5	1.6	2.4	2.3	2.3
	Bc0467	2.1	1.7	1.5	0.9	2.5	2.6	2.5

Table S7: Quality indices for the constructed PDA models

Model	Quality Index			Z-score	RMSD (Cα) from Template for aligned region	C-score	TM-score	QMEAN
	PDA template for best model	N aligned res	Seq. identity %					
Ba1977	Bc1974	206	99	3.46	0.4	-1.21	0.56	-0.50
Bc2929	Bc1974	206	81	3.97	0.4	-1.31	0.69	-1.17
Ba3679	Ba0150	198	36	3.81	0.9	-2.79	0.87	-1.84
Bc0171	Ba0150	206	95	3.48	0.5	-0.66	0.63	0.58
Bc0467	Ba0424	203	99	4.39	2.4	1.66	0.85	-1.70
Ba5436	Bc1974	206	41	3.87	0.6	0.82	0.82	-0.78

The confidence of each model is quantitatively measured by score indices that are calculated based on the significance of threading template alignments and the convergence parameters of the structure assembly simulations. **Z-score** is the score of the threading alignments. Alignment with a Normalized Z-score >1 mean a good alignment. **C-score** is a confidence score for estimating the quality of predicted models by I-TASSER. C-score is typically in the range of (-5, 2), where a C-score of a higher value signifies a model with a higher confidence. **RMSD** is an average distance of all residue pairs (Cα) in two structures. **TM-score** is a proposed scale for measuring the structural similarity between two structures. A TM-score >0.5 indicates a model of correct topology and a TM-score < 0.17 means a random similarity [87].

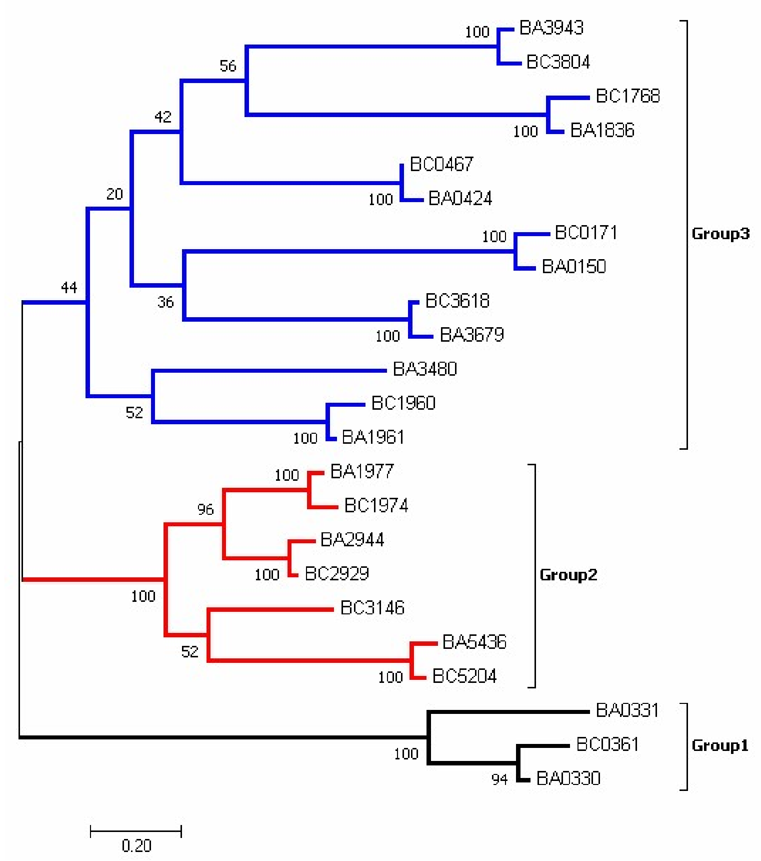

Figure S1. Molecular Phylogenetic analysis of the *B. anthracis* (BA) and *B. cereus* (BC) PDA Nodb domain nucleotide sequences by the Maximum Likelihood method based on the General Time Reversible model [1].The tree with the highest log likelihood (-6638.57) is shown. The percentage of trees in which the associated taxa clustered together is shown next to the branches. Initial tree(s) for the heuristic search were obtained automatically by applying Neighbor-Join and BioNJ algorithms to a matrix of pairwise distances estimated using the Maximum Composite Likelihood (MCL) approach, and then selecting the topology with superior log likelihood value. A discrete Gamma distribution was used to model evolutionary rate differences among sites (5 categories (+*G*, parameter = 2.5284)). Codon positions included were 1st+2nd+3rd+Noncoding. All positions containing gaps and missing data were eliminated. There were a total of 390 positions in the final dataset. The unrooted tree is drawn to scale, with branch lengths measured in the number of substitutions per site. The sequences are clustered in three groups represented with different colors accordance with figure 2a.

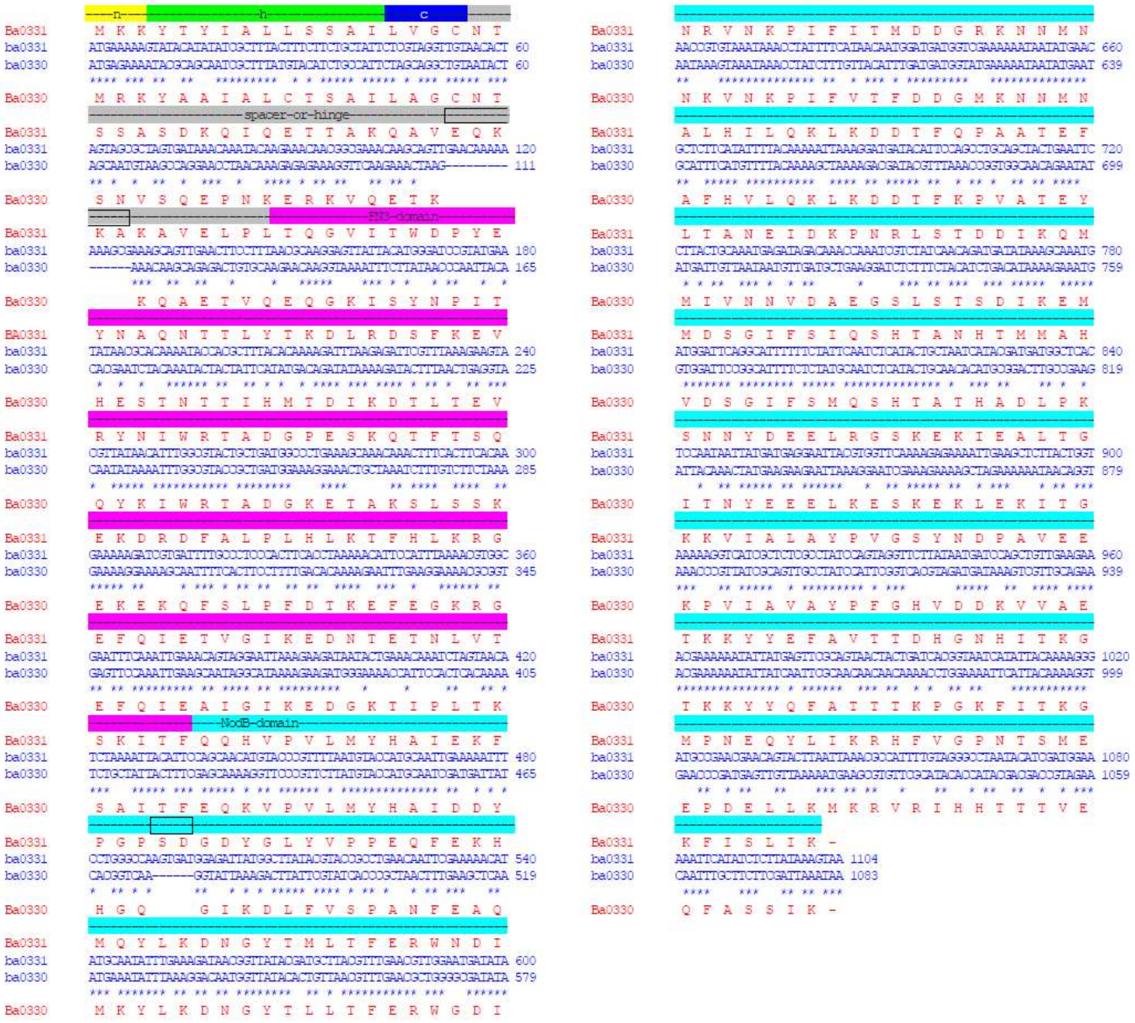

Figure S2. Sequence alignment between Ba0330 and Ba0331. The n- (in yellow), h- (in green) and c- (in blue) regions of the signal peptide predicted by the DOLOP –database are shown. The helical hinge region (in grey), the Fn3 domain (in magenta) and the NodB domain (in cyan) are also shown. The two insertions (depicted boxes) in Ba0331 are located in the helical hinge and in the β9-β10 loop of the NodB domain, respectively. Nucleotide sequences (in blue) and protein sequences (in red) are quoted. Alignment was performed using ClustalO.

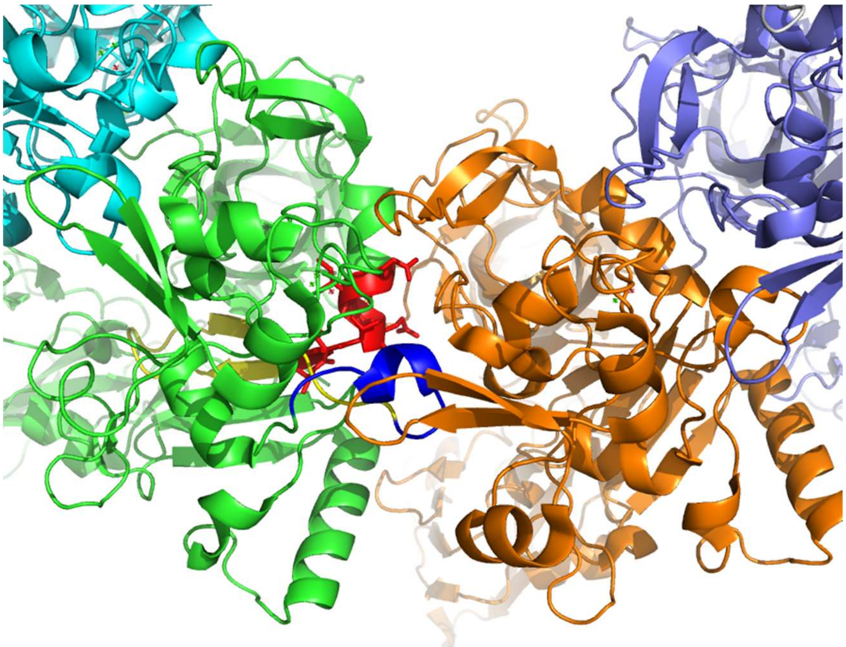

Figure S3. The QIQETTA α-helix forming sequence (in red) present in the oligomeric translocase channel formation interface of the D2 domain of the protective antigen (PA) (shown in green ribbon representation), an anthrax toxin component responsible together with the LF and EF for the virulence of *B. anthracis.* Both the QIQETTA helix (in red) and the membrane insertion loop (MIL) (in blue) [88] interact with the adjacent PA subunit (in orange) of the octamer. Diagram showing part of the octameric assembly of the protective antigen component of anthrax toxin (PDB Code: 3HVD) was made using PyMOL.

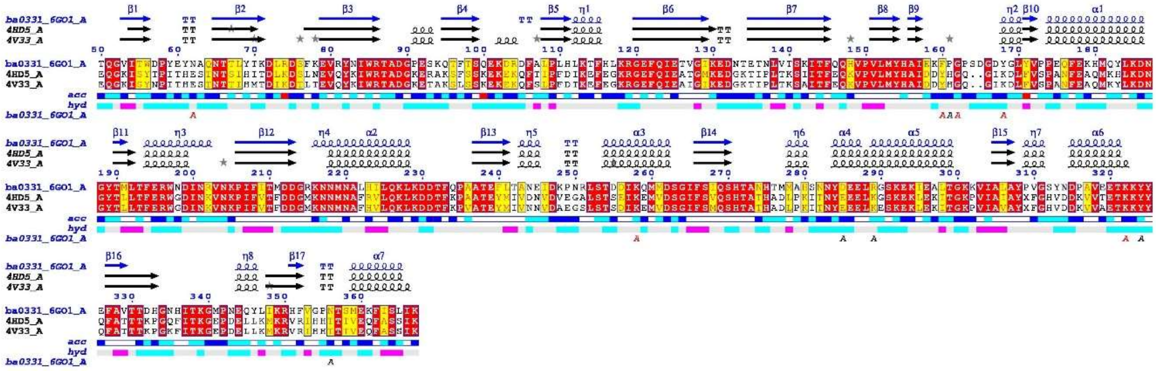

Figure S4. Conservation observed within Group1 structures. The totally conserved residues are shown in red and the partially conserved are shown in yellow in accordance to figure 6b and 7a. Secondary structure elements for the three structures are given on top and solvent accessibility and hydropathy scales per residue are given on bottom. Diagram drawn with ENDSCRIPT.

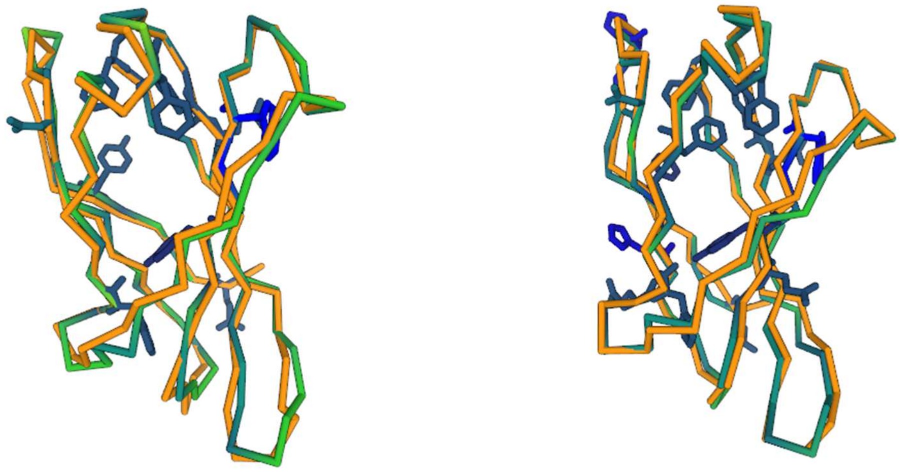

A **B**

Figure S5. Superimposition of the Fn3 structural domains of: (**a**) Ba0330 (orange backbone) and Ba0331 (green backbone) with Ca backbone RMS calculated at 1.1 Å and (**b**) Ba0330 (orange backbone) and Bc0361 (green backbone) with Ca backbone RMS at 0.9 Å. In blue sticks the conserved residues are shown. Diagrams are drawn using server DALI.

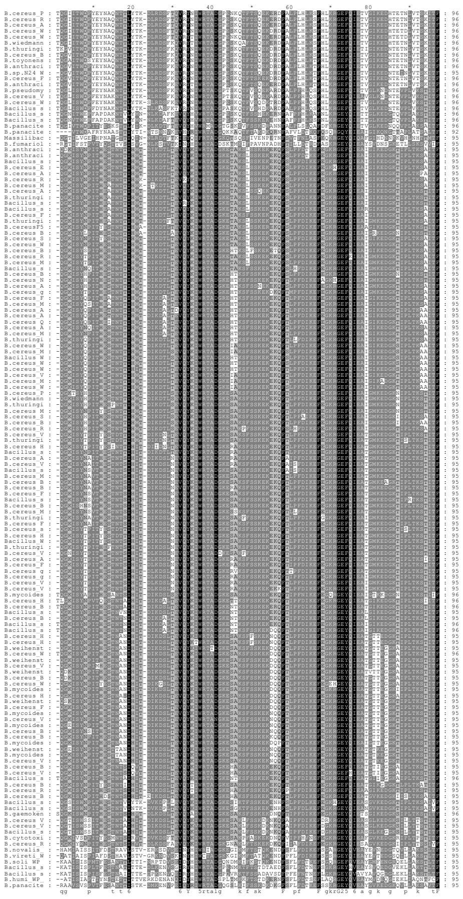

a

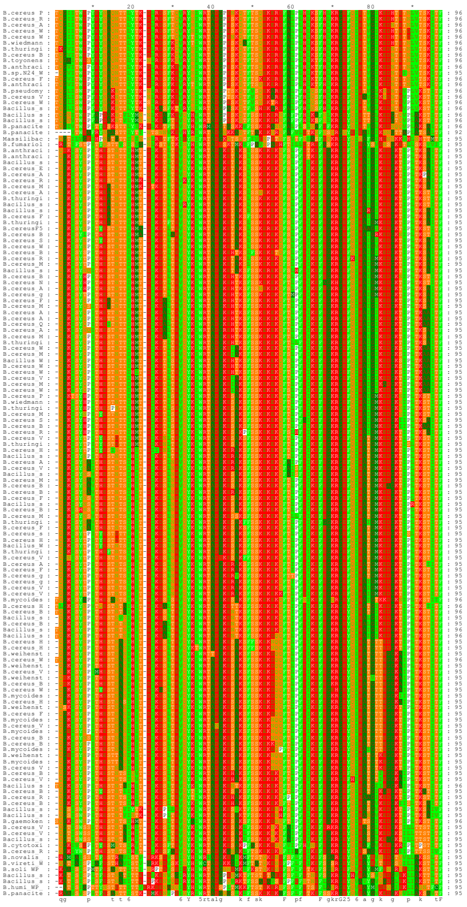

B

Figure S6. Multiple sequence alignment of Fn3 domains from Bacilli PDA sequences. (a) The intensity of color in greyscale indicates the degree of conservation across the alignment (black for highly conserved residues and grey levels for partially conserved ones.). (b) Residues are colored according to their physicochemical properties. Charged (Asp, Glu, Arg, Lys, His) in red, hydrophobic (Leu, Ile, Val, Phe, Tyr, Trp) in light green, polar (Ser, Thr) in orange, small residues (Ala, Gly) in dark green and Proline in white. Pictures created using GENEDOC.

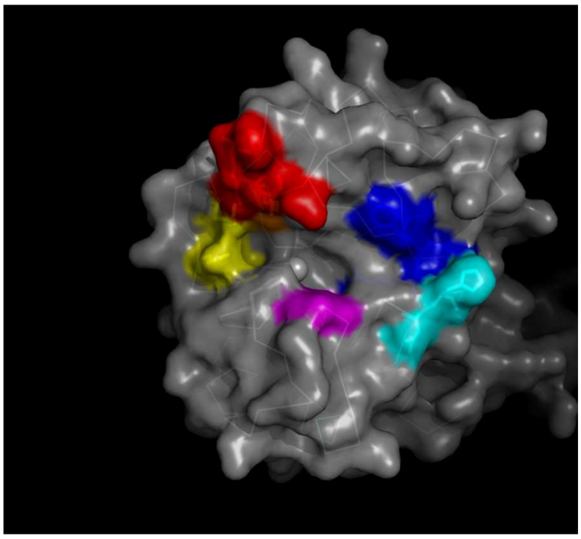

Ba0331

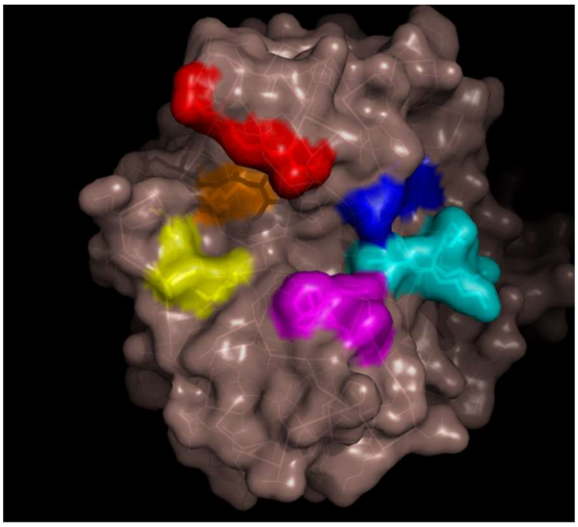

Ba0330

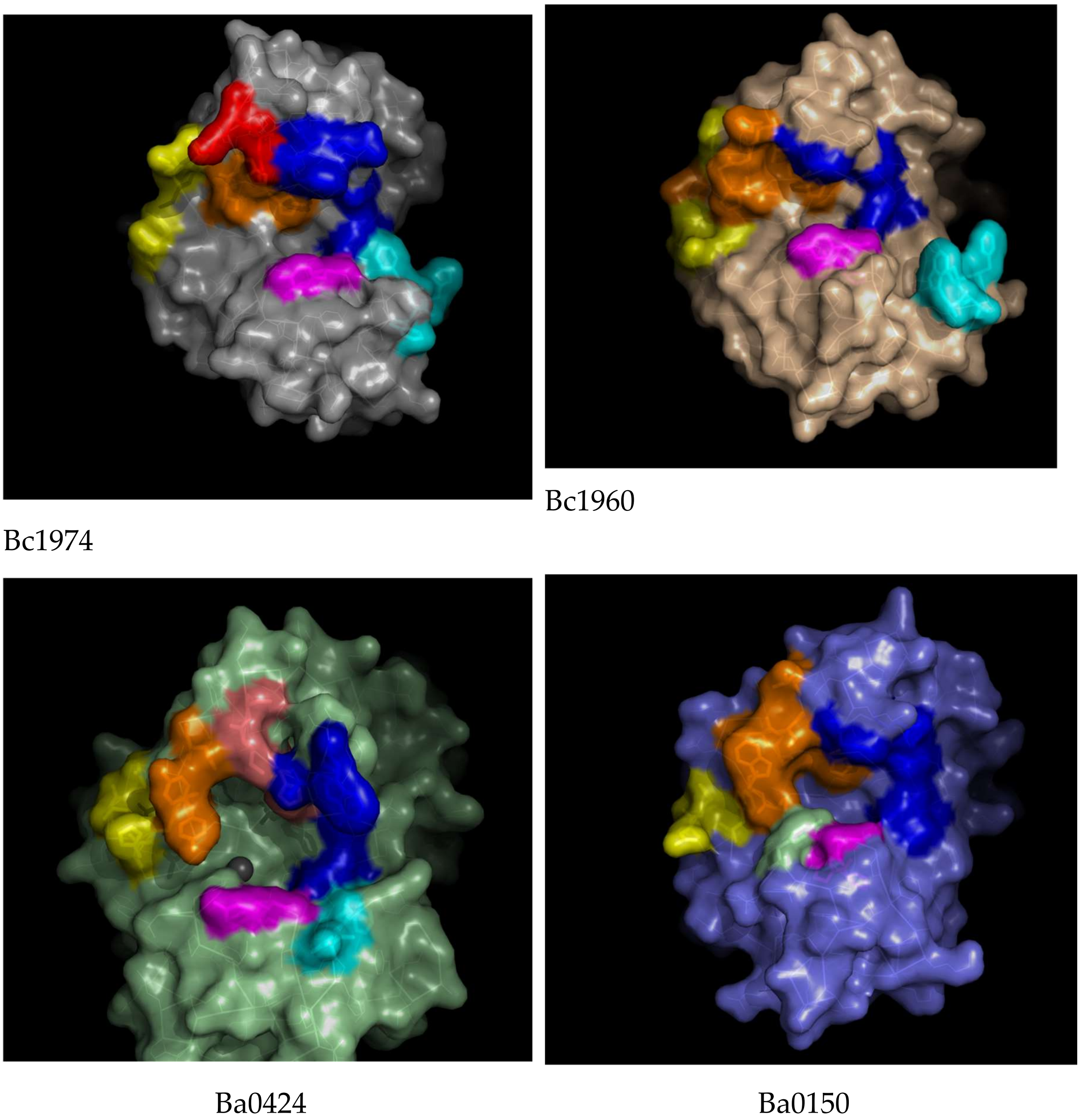

Figure S7. Comparison of NodB PDAs binding sites for *B. anthracis* and *B. cereus* structures. Surface representation diagrams of Ba0331, Ba0330, Bc1974, Bc1960, Ba0424 and Ba0150 NodB binding domain. The five sequence motifs forming the binding site are colored differently as shown in the sequence alignment in Figure 4. The MT3 motif (shown in magenta) is conserved in position while the others vary in position and composition.

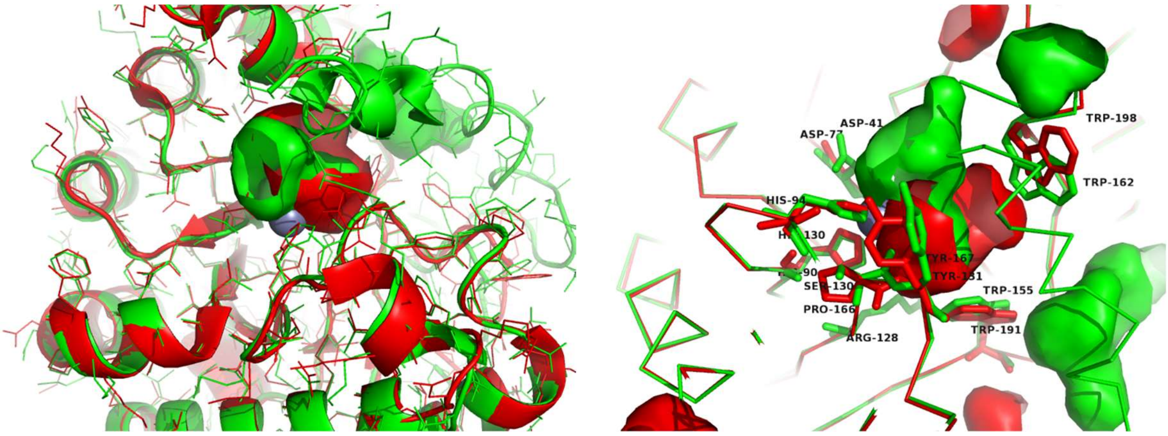

a B

Figure S8. Superposition of the NodB domain of the constructed model of Bc2929 (in green) on the Bc1974 structure (in red) (**a**). Overview of the backbone structure in ribbon representation with side chains in line representation and active site volume (**b**). Close-up on the binding site with backbone in line representation and binding site forming residues in stick representation. The metal ion is represented with a grey sphere.

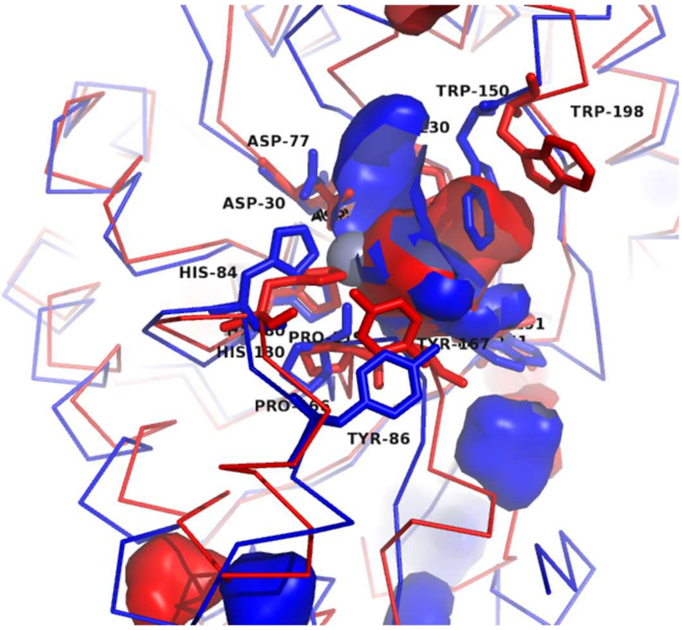

Figure S9. Superposition of the NodB domain of the constructed model of Ba3679 (in blue) on Bc1974 (in red). Close-up on the binding site with backbone in line representation and binding site forming residues in stick representation. The metal ion is represented as a grey sphere. Surfaces represent the available volume in the binding site.

References

1. Zhang, Y.; Skolnick, J. Scoring function for automated assessment of protein structure template quality. *Proteins*, **2004**, *57*, 702–710, doi:10.1002/prot.20264.
2. Kintzer, A.F.; Thoren, K.L.; Sterling, H.J.; Dong, K.C.; Feld, G.K.; Tang, I.I.; Zhang, T.T.; Williams, E.R., Berger, J. M.; Krantz, B. A. The protective antigen component of anthrax toxin forms functional octameric complexes. *J. Mol. Biol.* **2009**, *392*, 614–629, doi:10.1016/j.jmb.2009.07.037.
